# GRP75 triggers white adipose tissue browning to promote cancer-associated cachexia

**DOI:** 10.1038/s41392-024-01950-w

**Published:** 2024-09-26

**Authors:** Xu Chen, Qingnan Wu, Wei Gong, Shaolong Ju, Jiawen Fan, Xiaohan Gao, Xingyang Liu, Xiao Lei, Siqi Liu, Xiangdong Ming, Qianyu Wang, Ming Fu, Yongmei Song, Yan Wang, Qimin Zhan

**Affiliations:** 1 State Key Laboratory of Molecular Oncology, National Cancer Center/National Clinical Research Center for Cancer/Cancer Hospital, Chinese Academy of Medical Sciences and Peking Union Medical College, 100021 Beijing, China; 2 Key Laboratory of Carcinogenesis and Translational Research (Ministry of Education/Beijing), Laboratory of Molecular Oncology, Peking University Cancer Hospital & Institute, 100142 Beijing, China; 3 Peking University-Yunnan Baiyao International Medical Research Center, 100191 Beijing, China; 4 Center for Infection and Immunity, The Fifth Affiliated Hospital of Sun Yat-sen University, Zhuhai, China; 5 Department of Biochemistry and Biophysics, School of Basic Medical Sciences, Peking University Health Science Center, Beijing, China; 6 Research Unit of Molecular Cancer Research, Chinese Academy of Medical Sciences, Beijing, China; 7 Soochow University Cancer Institute, Suzhou, 215000 China

**Keywords:** Cancer, Cell biology

## Abstract

Cachexia, which affects 50–80% of cancer patients, is a debilitating syndrome that leads to 20% of cancer-related deaths. A key feature of cachexia is adipose tissue atrophy, but how it contributes to the development of cachexia is poorly understood. Here, we demonstrate in mouse models of cancer cachexia that white adipose tissue browning, which can be a characteristic early-onset manifestation, occurs prior to the loss of body weight and skeletal muscle wasting. By analysing the proteins differentially expressed in extracellular vesicles derived from cachexia-inducing tumours, we identified a molecular chaperone, Glucose-regulated protein 75 (GRP75), as a critical mediator of adipocyte browning. Mechanistically, GRP75 binds adenine nucleotide translocase 2 (ANT2) to form a GRP75–ANT2 complex. Strikingly, stabilized ANT2 enhances its interaction with uncoupling protein 1, leading to elevated expression of the latter, which, in turn, promotes adipocyte browning. Treatment with *withanone*, a GRP75 inhibitor, can reverse this browning and alleviate cachectic phenotypes in vivo. Overall, our findings reveal a novel mechanism by which tumour-derived GRP75 regulates white adipose tissue browning during cachexia development and suggest a potential white adipose tissue-centred targeting approach for early cachexia intervention.

## Introduction

Cachexia, which originated from the Greek words ‘*kakos*’ and ‘*hexis*’, meaning ‘bad state’, is one of the main complications in patients with cancer and is a fatal wasting syndrome affecting 50–80% of cancer patients, with the highest prevalence of digestive system tumours.^1,2^ This systemic condition characterized by systemic inflammation, skeletal muscle and adipose tissue wasting and, in turn, unintended body weight loss is often associated with anorexia and additional energy consumption, accounting for approximately 20% of cancer-related deaths,^2^ as well as subsequent intolerance to anticancer therapy.^3^ In clinical practice, cachexia is considered a continuum with three diagnostic stages: precachexia, cachexia, and refractory cachexia.^1,4^ Once patients reach the refractory stage, they are in a state of severe physical decline, characterized by aggressive hyper-catabolism and poor response to medications, making effective weight loss management challenging. Therefore, systematic exploration of early physical alterations to elucidate the molecular mechanism involved in cachexia initiation and effective interventions to halt the progression of cachexia are pressing issues to be addressed in the field of cachexia research.

Cachexia affects diverse tissues beyond muscle, including white adipose tissue (WAT).^5^ The extent of decrease in skeletal muscle, subcutaneous fat, and visceral fat area on computed tomography images from initial diagnosis to recurrence is positively correlated with a poorer prognosis following the recurrence of non-small cell lung cancer, indicating the potential utility of body composition in prognostic prediction.^6^ In particular, metabolic and morphological changes in WAT are already detected during early cachexia development and cause wasting,^7,8^ which often contributes to muscle loss.^9^ Additionally, the degree of WAT loss has been associated with survival and prognosis in cancer patients with cachexia, highlighting the contribution of WAT alterations to cachexia progression.^10,11^ In mammals, adipose tissue not only is a lipid-storage and secretory tissue but also plays a vital role in regulating whole-body energy homoeostasis. There are two main types of adipose tissue, WAT and brown adipose tissue (BAT), each of which possesses distinct morphological, functional, and metabolic properties.^12^ Unlike BAT, which is a site for energy dissipation and thermogenesis, WAT, which accounts for 15–40% of human body weight, has long been recognized as a vast depot of lipids, primarily in the form of triglycerides (TGs).^12^ Cachectic adipose tissue atrophy results from enhanced TG degradation (lipolysis) and decreased lipid synthesis (lipogenesis).^13^ These lipolytic products, fatty acids and glycerol can be re-esterified in adipose tissue, creating an energy-consuming futile substrate cycle.^14^ Both of these pathways could contribute to WAT loss during cancer cachexia progression. Recently, another vital catabolic pathway, the conversion from WAT to a BAT-like phenotype called “WAT browning”, has been observed in mice and patients with various wasting conditions, including cancer, and has been implicated in cachexia-induced WAT atrophy.^15–17^ Accordingly, browned WAT adopts multilocular lipid droplets and expresses thermogenic genes, such as uncoupling protein 1 (UCP1), which is located in the inner membrane of the mitochondria and dissipates energy to enhance thermogenesis.^18^ Additionally, overexpression of UCP1 has been observed in subcutaneous WAT of patients with early-stage pancreatic ductal adenocarcinoma,^8^ suggesting that WAT browning might be a critical mechanism of WAT atrophy in early cachexia development. However, the precise mechanisms underlying the remodelling of WAT during cachexia initiation remain elusive and require further investigation.

Tumour cells can communicate and impact surrounding cells or distant organs by secreting extracellular vesicles (EVs).^19^ EVs are membranous vesicles containing nucleic acids, various metabolites, and proteins that are stably present in biological fluids and mediate inter-tissue communication in pathological and physiological situations.^20^ EVs are increasingly recognized as contributing factors in a range of diseases, including cancer, and their contents can be leveraged to investigate pathology and serve as diagnostic biomarkers,^19^ which is further supported by the evidence that inhibiting the production of tumour-derived EVs can ameliorate cancer cachexia-associated wasting.^21,22^ Several previous studies have advanced knowledge regarding the involvement of tumour-derived EVs in distant tissue dysfunction.^23,24^ For instance, EVs associated with heat shock proteins 70 and 90 (Hsp70/90) released by cancer cells that induce cachexia have been shown to promote muscle wasting through the activation of Toll-like receptor 4 (TLR4).^23^ However, the mechanisms by which factors derived from tumour-derived EVs induce adipocyte browning and atrophy remain poorly understood.

In the present study, by setting three time points (T-1 to T-3), we systematically depicted the dynamic alterations in physical parameters and revealed cancer-induced WAT dysfunction in our established esophageal squamous cell carcinoma (ESCC)-induced cachexia mouse model.^24^ The current study aimed to identify novel extracellular vesicular proteins derived from cachexia-inducing tumours and elucidate the underlying mechanism involved, thereby expanding our understanding of their contribution to the aetiology of cancer-induced WAT dysfunction and exploring their potential for clinical application.

## Results

### WAT atrophy precedes muscle wasting during early cachexia development in tumour-bearing mice

**Fig. 1:**
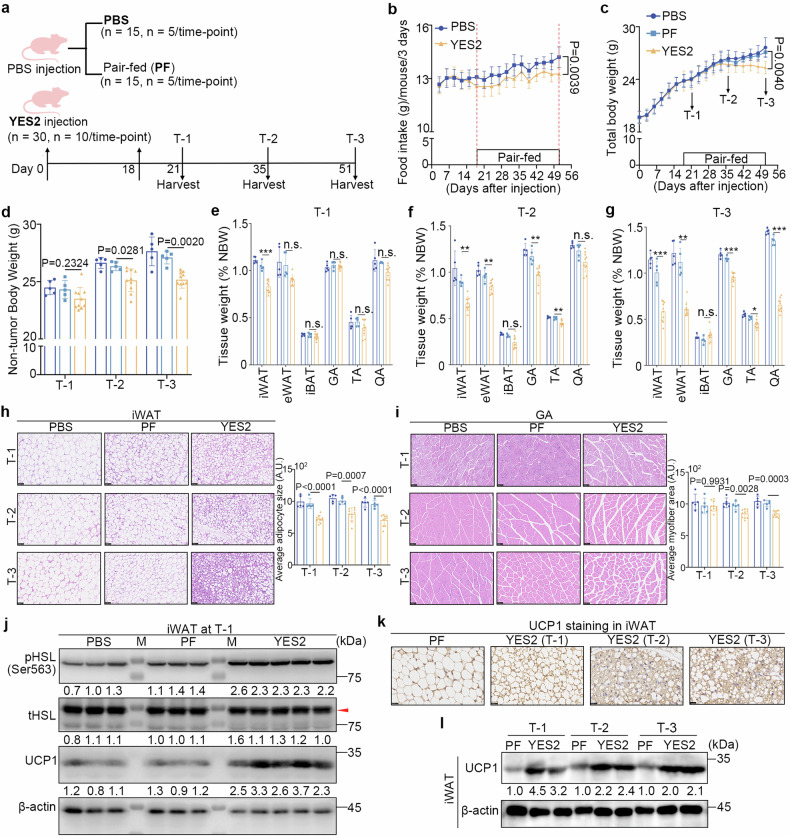
WAT atrophy precedes skeletal muscle wasting during early cachexia development in YES2 tumour-bearing mice. **a** Schematic diagram of in vivo subcutaneous injection of PBS or YES2 cells in the right flank of six-week-old BALB/C nude mice and corresponding samples collected at three time points (T-1, T-2, and T-3). The pair-fed (PF) group was established on day 18 post inoculation. **b** Food intake curves of the PBS and YES2 groups. **c** Total body weight (TBW) curves of the three groups. **d** Non-tumour body weight (NBW) of the three groups at three time points. **e**–**g** Ratios of tissue weight to NBW, including adipose tissues (iWAT, eWAT, and iBAT) and skeletal muscle (GA, TA, and QA), in the three groups at T-1 (**e**), T-2 (**f**), and T-3 (**g**). **h**, **i** Representative hematoxylin and eosin (H&E) staining images of iWAT (**h**) and GA (**i**) from the three groups at three time points. Scale bar, 50 μm. The quantified adipocyte sizes and cross-sectional areas of the myofibers are shown on the right. **j** Immunoblots of p-HSL (Ser563), total HSL, and UCP1 in iWAT from the three groups at T-1. M: molecular mass markers. **k** Representative immunohistochemistry (IHC) images of UCP1 staining in iWAT from YES2 groups at three time points; iWAT from the PF group was used as a negative control. Scale bar, 25 μm. **l** Immunoblots of UCP1 in the iWAT of the PF and YES2 groups at three time points. The data are presented as the mean ± SEM (*n* = 5 for the PBS and PF groups; *n* = 10 for the YES2 group at each time point). The exact *P* values were tested with an unpaired two-tailed Student’s *t* test (**b**), a multiplied *t* test (**c**), and one-way analysis of variance (ANOVA) (**d**–**i**). *P < 0.05; **P < 0.01; ***P < 0.001; n.s. no significance, iWAT inguinal white adipose tissue, eWAT epididymal WAT, iBAT interscapular brown adipose tissue, GA gastrocnemius, TA tibialis anterior, QA quadriceps femoris

To characterize the dynamic changes and mechanisms of early cachexia development, we monitored the alterations in food consumption, total body weight (TBW), non-tumour body weight (NBW), and body composition in the ESCC-induced cachexia mouse model^24^ by establishing three time points, T-1, T-2, and T-3, as shown in Fig. 1a. In agreement with the frequent occurrence of anorexia in cachexia,^25^ a reduction in food intake was observed from day 18 post tumour inoculation in YES2 tumour-bearing mice (YES2 group) compared to that in tumour-free mice (PBS group) (Fig. 1b). To eliminate the interference of food intake, an age- and sex-matched non-tumour-bearing group was pair-fed with the YES2 group (PF group). Due to tumour growth (Supplementary Fig. 1a–b), the 6.6% decrease in TBW of the YES2 group at T-3 in comparison with that of the PF group was in agreement with clinical cachexia features (weight loss ≥5%), as shown in Fig. 1c. Despite no significant difference, the TBW in the YES2 group decreased from 0.6% to 2% at T-1 and T-2 compared to that in the corresponding PF groups (Fig. 1c), which coincided with the clinical phenotypes of the pre-cachexia stage (body weight loss <5% with anorexia). The detection of NBW revealed a significant decrease in the YES2 group at T-3, with a 7% weight loss relative to the corresponding PF group (Fig. 1d). Notably, there was a 3.3% to 4.6% decrease in the NBW of the YES2 group from T-1 to T-2 (Fig. 1d), indicating that alterations in body composition occur during the early stages of cachexia progression.

Weight loss associated with cachexia is characterized by muscle and adipose tissue wasting.^5^ However, the specific tissues that initiate cachectic phenotypes remain unclear. To this end, the ratios of skeletal muscle and adipose tissues to the corresponding NBW were measured at three time points. Impressively, at T-1, both the ratios of inguinal WAT (iWAT; a subcutaneous fat depot) and epididymal WAT (eWAT; a form of visceral fat) in the YES2 group decreased, whereas the ratio of interscapular BAT (iBAT) did not significantly change (Fig. 1e). The ratios of skeletal muscles, including the gastrocnemius (GA), tibialis anterior (TA), and quadriceps femoris (QA), showed no differences between the YES2 and PF groups (Fig. 1e). These phenotypes, together with decreased food intake at T-1 in the absence of TBW loss, were defined as the “super pre-cachexia stage” in this study. At T-2, the YES2 group exhibited steady WAT atrophy and a notable reduction in skeletal muscle/NBW (Fig. 1f), together with 2% TBW loss, which are consistent with the clinical features of pre-cachexia (weight loss <5%). At T-3, YES2 tumour-bearing mice showed cachexia phenotypes, exhibiting more than 5% weight loss with ongoing WAT and skeletal muscle wasting (Fig. 1g).

Alterations in the WAT and skeletal muscle during the development of cachexia were also confirmed by morphological and histological analyses (Fig. 1h–i and Supplementary Fig. 1c). Since systemic inflammation is recognized as another feature of both experimental and clinical cachexia,^15,26^ the serum levels of interleukin 6 (IL-6) and tumour necrosis factor α (TNF-α) were measured. IL-6 levels gradually increased with cachexia progression in the YES2 group (Supplementary Fig. 1d). TNF-α levels were also upregulated but not significantly in the YES2 group compared to the PF group (Supplementary Fig. 1e). These results demonstrated the presence of systemic inflammation during the progression of cachexia. Overall, YES2 tumour-bearing mice exhibited cachectic features that support clinical cachexia development, with a notable emphasis on early signs of WAT atrophy.

To investigate the pathogenesis of WAT atrophy in the early stage of ESCC-induced cachexia development, the expression of HSL, a key enzyme for lipolysis,^15^ and UCP1, a marker protein for browning,^15^ in WAT in a mouse model was examined. Immunoblot analysis indicated a 2.9-fold increase in UCP1 abundance in the iWAT of the YES2 group at T-1 compared to that in the PF group, accompanied by a 2.3-fold increase in phosphorylated hormone-sensitive lipase (HSL) expression (Fig. 1j). Notably, immunohistochemical analysis revealed that UCP1-positive adipocytes were present throughout the development of cachexia from T-1 to T-3, prominently in the super pre-cachexia stage (T-1) in the YES2 tumour-bearing mice compared to their PF counterparts (Fig. 1k), which was supported by immunoblotting (Fig. 1l). The expression of other browning-related proteins,^9^ peroxisome proliferator-activated receptor-γ coactivator-1α (PGC1α), was found to be upregulated, whereas the expression of cell death-induced DFFA-like effector A (CIDE-A) was not (Supplementary Fig. 1f). Additionally, there was no significant difference in the mRNA levels of the muscle-specific E3 ligases *Murf-1* and *Atrogin-1*, which are two markers of muscle wasting,^27^ among the three groups at T-1 (Supplementary Fig. 1g). However, these genes were significantly upregulated in YES2 tumour-bearing mice at T-2 and T-3 (Supplementary Fig. 1g). These results indicate that WAT browning may act as an initial symptom of cachexia, leading to WAT atrophy in ESCC-induced cachectic immunodeficient mice.

To expand the findings to normal immune conditions, mouse-origin Lewis lung carcinoma (LLC) cells were used to induce cachexia in C57BL/6 J mice (Supplementary Fig. 2a). Although the TBW of LLC tumour-bearing mice increased due to tumour growth (Supplementary Fig. 2b–d), the NBWs decreased substantially compared to that in the corresponding PF groups (Supplementary Fig. 2e). Progressive adipose and muscle loss was observed in the LLC groups (Supplementary Fig. 2f). Notably, the observation that WAT browning occurred during early cachexia development was also confirmed in this model (Supplementary Fig. 2g–i).

These data highlight that WAT browning is an important initial event and persists during cachexia progression in mice with, and is not limited to, ESCC.

### Extracellular vesicles derived from YES2 cells stimulate adipocyte browning in vitro

**Fig. 2:**
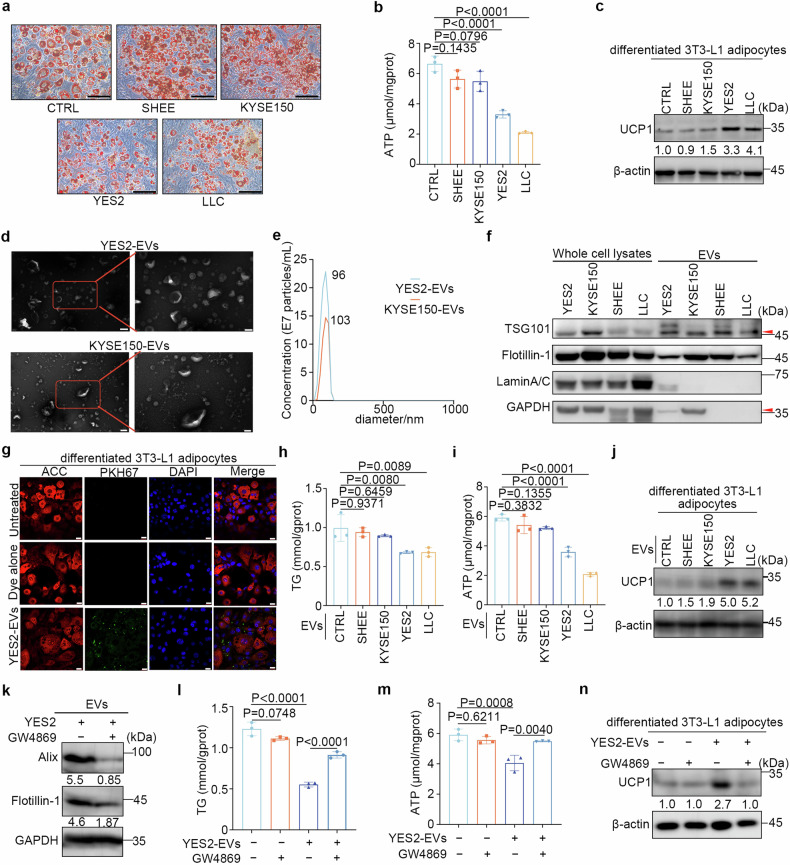
Extracellular vesicles derived from YES2 cells stimulate adipocyte browning in vitro. **a** Representative Oil Red O staining images of differentiated 3T3-L1 adipocytes cocultured with SHEE, KYSE150, YES2, and LLC cells. Untreated 3T3-L1 adipocytes (CTRL) are shown as a negative control. Scale bar, 100 μm. **b** Quantitation of adenosine 5’-triphosphate (ATP) content in differentiated 3T3-L1 adipocytes as described in **a**. **c** Immunoblots of UCP1 in adipocytes treated as described in **a**. **d** Representative transmission electron microscope images of YES2- and KYSE150-derived EVs. Scale bar, 200 nm (left); 100 nm (right). **e** Nanoparticle tracking of the size and quantity of YES2-EVs and KYSE150-EVs. **f** Immunoblots of protein markers in whole lysates of cells (GAPDH and Lamin A/C) and corresponding EVs (TSG101 and flotillin-1). **g** Immunofluorescence staining of differentiated 3T3-L1 adipocytes incubated with PKH67-labelled YES2-EVs. Green, PKH67; red, acetyl-CoA carboxylase (ACC); blue, DAPI. Scale bar, 20 μm. **h**, **i** Quantitation of intracellular triglyceride (TG) (**h**) and ATP (**i**) levels in differentiated 3T3-L1 adipocytes treated with SHEE-EVs, KYSE150-EVs, YES2-EVs, or LLC-EVs. **j** Immunoblots of UCP1 in differentiated 3T3-L1 adipocytes as described in **h**. **k** Immunoblots of Alix and flotillin-1 in EVs secreted by YES2 cells pretreated with DMSO or GW4869 (10 μM). **l**, **m** Quantitation of TG (**l**) and ATP (**m**) levels in differentiated 3T3-L1 adipocytes treated with DMSO, GW4869, YES2-EVs or EVs obtained from GW4869-treated YES2 cells. Untreated adipocytes are shown as a negative control. **n** Immunoblots of UCP1 in differentiated 3T3-L1 adipocytes treated as described in **l**. The data are presented as the mean ± SEM. The exact *P* values were tested with one-way ANOVA in (**b**, **h**, **i**, **l**, and **m**)

Browning in WAT is a process from an energy-storing state to an energy-depleting state, often accompanied by a reduction in intracellular lipid droplets and increased energy dissipation and thermogenic gene expression.^18^ To explore how ESCC contributes to WAT browning, we cocultured adipocytes with tumour cells and found that the cachexia-inducing cell lines YES2 and LLC led to a marked reduction in lipid droplet size (Fig. 2a) and adenosine triphosphate (ATP) content (Fig. 2b) in adipocytes compared to those of the noncell coculture control. In contrast, incubation with SHEE or a non-cachexia inducing cell line, KYSE150,^24^ failed to induce such changes. Notably, the abundance of UCP1 protein was also 3.3-fold and 4.1-fold greater in adipocytes cocultured with YES2 and LLC cells, respectively (Fig. 2c), demonstrating that YES2-derived factors can promote cachectic adipocyte browning.

Tumour-derived EVs have been found to carry various signalling molecules to target recipient cells, thereby regulating the induction of cachexia-associated wasting.^22,24^ To examine whether ESCC-derived EVs could stimulate adipocyte browning, YES2-EVs and KYSE150-EVs were isolated using standard methods, and electron microscopy, NanoSight, and immunoblotting were performed to identify their structures, sizes, and protein markers (Fig. 2d–f). The increased accumulation of PKH67-labelled EVs in adipocytes suggested that YES2-EVs could be internalized by adipocytes (Fig. 2g). Consistently, YES2-EVs, as well as LLC-EVs, caused a noticeable decrease in the intracellular TG and ATP contents of adipocytes, whereas neither SHEE-EVs nor KYSE150-EVs did (Fig. 2h–i). The UCP1 expression was increased 5.0- and 5.2-fold in adipocytes treated with YES2-EVs and LLC-EVs, respectively (Fig. 2j). To verify the functional significance of tumour-derived EVs, YES2 cells were treated with the neutral sphingomyelinase inhibitor GW4869 to block the secretion of EVs (Fig. 2k). As expected, GW4869 treatment alleviated the decrease in TG and ATP levels and the upregulation of UCP1 in adipocytes induced by YES2-EVs (Fig. 2l–n). Taken together, these observations indicate that cancer cachectic WAT browning is induced by tumour-derived EVs.

### GRP75 is essential for adipocyte browning

**Fig. 3:**
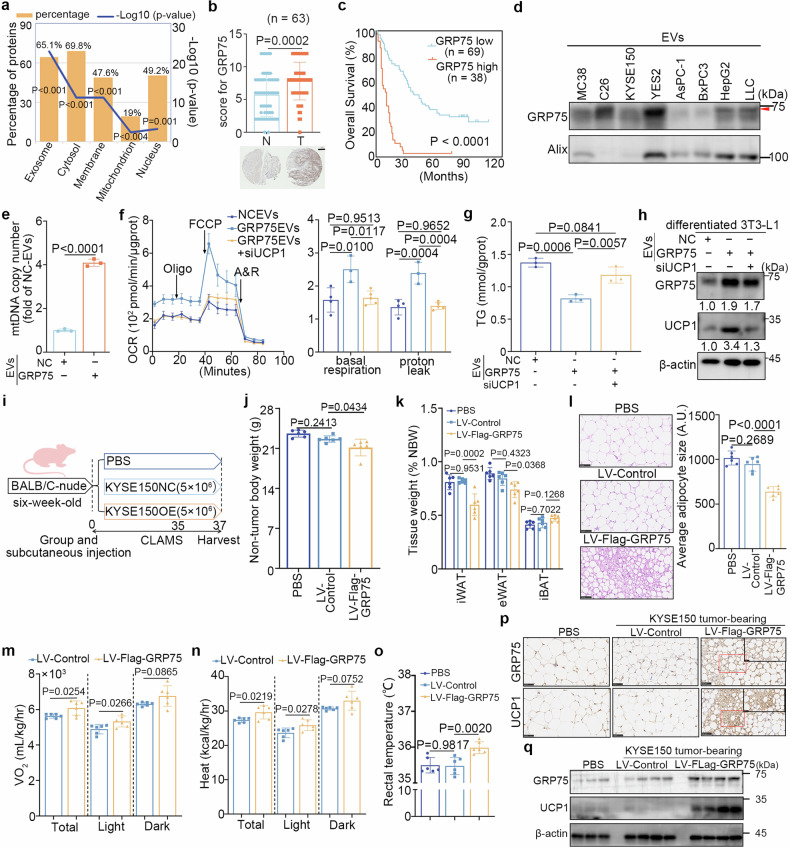
GRP75 is essential for adipocyte browning. **a** Five most representative subcellular localization analyses of 63 overlapping proteins. **b** Representative IHC images and the staining scores of GRP75 (top) in 63 pairs of patient tumours (T) and matched adjacent normal tissues (N). Scale bar, 200 μm. **c** Kaplan‒Meier survival analysis of patients with ESCC stratified by GRP75 expression (*n* = 107; *P* < 0.001, log-rank test). **d** Immunoblots of GRP75 in EVs derived from mouse colon cancer cells (MC38 and C26), ESCC cells (YES2 and KYSE150) and other cachexia-inducing tumour cells (HepG2, LLC, AsPC-1, and BxPC3). **e** MtDNA copy number was detected in differentiated 3T3-L1 adipocytes treated with NC-EVs or GRP75-EVs. **f** Oxygen consumption rate (OCR) in primary adipocytes treated by GRP75-EVs or GRP75-EVs with si-UCP1. Primary adipocytes treated with NC-EVs are shown as a negative control. Left: plot of the time course OCR normalized to the protein concentration. Right: calculated respiration levels of basal and proton leaked respiration. **g** Quantitation of intracellular TG contents in adipocytes treated as described in **f**. **h** Immunoblots of GRP75 and UCP1 in differentiated 3T3-L1 adipocytes treated as described in **f**. **i** Schematic diagram of in vivo subcutaneous injection of PBS, KYSE150 cells with stable GRP75 overexpression (LV-Flag-GRP75) or negative lentiviral vectors (LV-Control) in six-week-old BALB/c-nude mice (*n* = 6 per group). **j** NBWs of the PBS, LV-Control and LV-Flag-GRP75 groups. **k** Ratios of iWAT, eWAT, and iBAT to NBW in the three groups. **l** Representative H&E-stained images of iWAT from the three groups. Scale bar, 50 μm. Quantified adipocyte sizes are shown on the right side. **m**, **n** Oxygen consumption volume and heat production of the LV-Control and LV-Flag-GRP75 groups in the metabolic cage housed at 22°C (*n* = 6 per group). **o** Rectal temperatures of the PBS, LV-Control and LV-Flag-GRP75 groups. **p** Representative IHC images of UCP1 and GRP75 staining in iWAT from the three groups. Scale bar, 50 μm. The magnified image labelled with a red rectangle is shown on the upper right side. Scale bar, 25 μm. **q** Immunoblotting of GRP75 and UCP1 protein in iWAT from three groups (*n* = 4; representative of four biological replicates per group). The data are presented as the mean ± SEM. The exact *P* values were tested with one-way ANOVA in (**j**–**l**, **o**) and unpaired two-tailed Student’s *t* test in (**m** and **n**)

To identify potential factors contained in YES2-EVs that trigger adipocyte browning, the profiles of differentially expressed proteins between KYSE150-EVs and YES2-EVs were analysed using mass spectrometry (Supplementary Fig. 3a). Sixty-four proteins upregulated in YES2-EVs compared with KYSE150-EVs were identified by screening with ≥4 unique peptides in three independent and replicate experiments, 63 of which were identified in the Vesiclepedia database (Supplementary Fig. 3b). Surprisingly, gene ontology (GO) enrichment analysis revealed that nearly 20% of these proteins were classified as mitochondrial components (Fig. 3a), and almost half of these were heat shock proteins (HSP90, HSP70, GRP75, and GRP78), suggesting that tumour-secreted mitochondrial components may be associated with adipocyte browning. Immunoblots confirmed the high levels of 75-kDa glucose regulatory protein (GRP75), encoded by *Hspa9*, and UCP1 proteins in the iWAT of YES2 tumour-bearing mice from T-1 to T-3 (Supplementary Fig. 3c).

GRP75, a stress-induced molecular chaperone, belongs to the HSP70 family.^28^ It is traditionally localized in the mitochondria as an N-terminal signal peptide and regulates protein quality control, including the folding, assembly, and export of misfolded proteins for degradation.^28^ The overexpression of GRP75 in tumour tissues is often positively correlated with malignant progression.^29^ Proteomic analysis revealed that GRP75 was strongly associated with thermogenic adipocytes in perivascular adipose tissue,^30^ indicating the crucial role of GRP75 in tumour-induced adipocyte browning.

To investigate the clinical significance of GRP75, immunohistochemical assays were performed, and GRP75 expression was found to be substantially greater in 63 ESCC tissue samples than in corresponding adjacent normal tissue samples (Fig. 3b). Chi-square tests revealed a strong correlation between GRP75 expression and TNM stage (Supplementary Tables 1 and 2). Moreover, Kaplan–Meier survival analysis revealed that GRP75 overexpression was strongly associated with poor survival in patients with ESCC (Fig. 3c). Notably, the serum GRP75 concentration was substantially greater in the YES2 group than in the PF group, which corresponded to the progression of cachexia, especially WAT atrophy (Supplementary Fig. 3d). Immunoblot analysis revealed higher GRP75 levels in YES2-EVs than in EVs originating from KYSE150 (Fig. 3d). Additionally, GRP75 expression was enhanced in EVs secreted by other well-known cachexia-inducing cancer cells,^17,23^ including C26 (mouse colon cancer), HepG2 (human hepatocellular carcinoma), and LLC cells, relative to EVs from non-cachexia inducible cancer cell lines MC38 (mouse colon cancer) (Fig. 3d), suggesting that the upregulation of GRP75 is involved in pancancer cachexia.

To explore the molecular mechanism by which GRP75 regulates adipocyte browning, we stably overexpressed Flag-GRP75 in KYSE150 cells (LV-Flag-GRP75) and knocked down GRP75 transcription in YES2 cells (GRP75-KD) by infection with lentiviral vectors, and the corresponding EVs were isolated to treat adipocytes (Supplementary Fig. 3e, f). The activation of the browning process in adipocytes can lead to elevated expression of thermogenesis genes, such as UCP1, as well as the upregulation of oxygen consumption and mitochondrial biogenesis.^31^ Immunoblotting revealed increased levels of exogenous GRP75 in mitochondria isolated from adipocytes treated with GRP75-overexpressing EVs (Supplementary Fig. 3g), suggesting that tumour-derived GRP75 can be internalized by mitochondria. The administration of GRP75-overexpressing EVs increased mitochondrial biogenesis, as evidenced by an increase in mtDNA copy number (Fig. 3e); conversely, exposure to GRP75-KD EVs inhibited this increase (Supplementary Fig. 3h). Changes in the oxygen consumption rate (OCR) in adipocytes treated with GRP75-overexpressing EVs were therefore examined, and marked increases in the basal and proton leakage OCRs were detected (Fig. 3f). In contrast, GRP75-KD EVs substantially reduced the basal and proton leakage OCRs in adipocytes (Supplementary Fig. 3i). In parallel, TG levels were reduced in adipocytes treated with LV-Flag-GRP75 EVs, whereas TG levels were increased in adipocytes treated with GRP75-KD EVs (Fig. 3g and Supplementary Fig. 3j). Immunoblotting assays verified the elevated expression of UCP1 in adipocytes treated with GRP75-overexpressing EVs, whereas GRP7-KD EVs reversed these changes (Fig. 3h and Supplementary Fig. 3k). Furthermore, siRNA-mediated UCP1 knockdown ameliorated the decrease in the basal and proton leakage OCRs induced by GRP75 overexpression in adipocytes and enhanced the accumulation of intracellular TGs (Fig. 3f–h). These results suggest that GRP75-triggered adipocyte browning might be dependent on elevated UCP1.

To verify whether GRP75-induced browning is a general phenomenon in cancer cachexia, a GRP75-knockdown LLC cell line was established via si-GRP75 transfection (Supplementary Fig. 4a). As expected, EVs derived from GRP75-knockdown LLC cells inhibited adipocyte browning, as evidenced by the restoration of TG and ATP levels, reduction in mitochondrial biogenesis, and downregulation of UCP1 expression (Supplementary Fig. 4b–e). Upregulation of GRP75 was detected in the iWAT and serum of LLC tumour-bearing mice (Supplementary Fig. 4f–g), and there was an inverse correlation between the serum GRP75 concentration and the WAT/NBW ratio (Supplementary Fig. 4h, R^2^ = 0.5234, *P* < 0.0001). Collectively, these findings support that GRP75 plays a critical role in the pathogenesis of adipocyte browning in multiple cancer types.

To determine the role of elevated GRP75 expression in ESCC-induced WAT browning in vivo, BALB/c nude mice were subcutaneously inoculated with KYSE150 LV-Flag-GRP75 cells (Fig. 3i). Compared with those in mice injected with KYSE150 LV-Control cells, TBW and NBW in the LV-Flag-GRP75 group decreased by 6.5% and 6.6%, respectively (Supplementary Fig. 5a and Fig. 3j). There were no significant differences in food intake or tumour growth between the two groups (Supplementary Fig. 5b–d). The LV-Flag-GRP75 group exhibited decreased ratios of WAT to NBW, as opposed to skeletal muscle, as evidenced by decreased adipocyte sizes (Fig. 3k, l and Supplementary Fig. 5e, f). Additionally, the LV-Flag-GRP75 group displayed increased oxygen consumption and heat production, particularly during the light cycle, as well as increased rectal temperatures (Fig. 3m–o). Despite these metabolic changes, physical activity levels remained unchanged (Supplementary Fig. 5g). The induction of browning by GRP75 overexpression was validated through the upregulation of UCP1 (Fig. 3p, q). Furthermore, a twenty-fold increase in human GRP75 levels was detected in the serum of the LV-Flag-GRP75 group, with no notable differences in mouse GRP75, IL-6 or TNF-α levels among the three groups (Supplementary Fig. 5h–k). Taken together, these results suggested that GRP75 may serve as a crucial mediator of the tumour-induced browning phenotype in vivo.

### GRP75 binds to and stabilizes ANT2 by decreasing its ubiquitination level

**Fig. 4:**
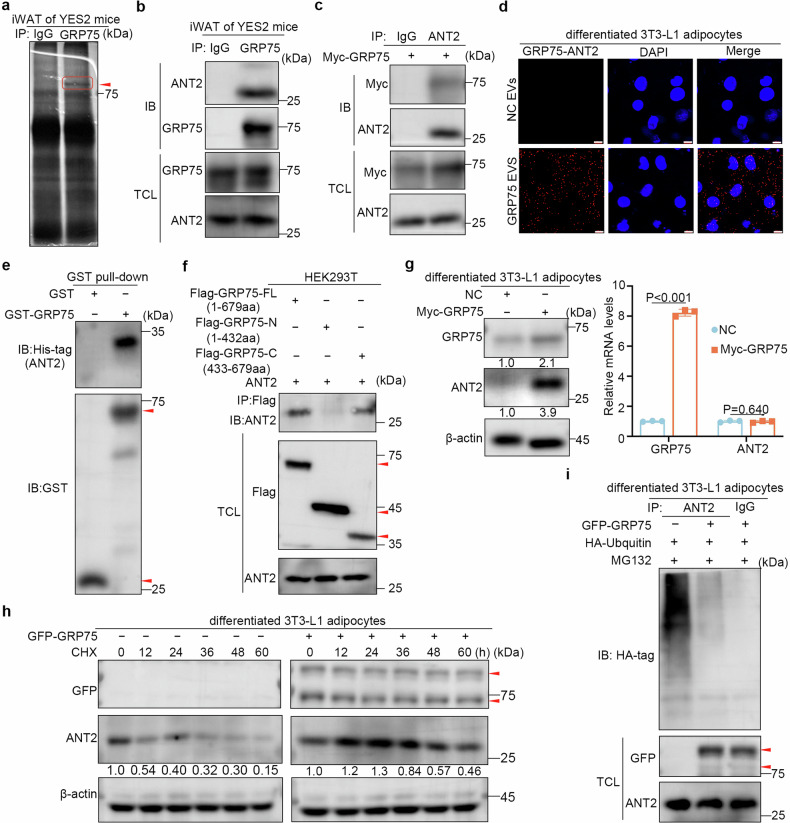
GRP75 binds to and stabilizes ANT2 by decreasing its ubiquitination level. **a** Identification of endogenous GRP75-interacting proteins. Extracts from the iWAT of YES2 tumour-bearing mice were incubated with protein A/G magnetic beads conjugated with an anti-GRP75 antibody. The eluted proteins were resolved by SDS‒PAGE and visualized by silver staining. The differential gel piece framed by the red rectangle was analysed by mass spectrometry. **b** Coimmunoprecipitation of endogenous ANT2 and GRP75 in the iWAT of YES2 tumour-bearing mice. Anti-IgG was used as a negative control. **c** Coimmunoprecipitation of GRP75 and ANT2 in differentiated 3T3-L1 adipocytes transfected with Myc-GRP75 plasmids. **d** PLA signals and quantification of the combination of anti-GRP75 and anti-ANT2 in differentiated 3T3-L1 adipocytes treated with NC-EVs or GRP75-overexpressing EVs. DAPI, nuclei. Scale bar, 10 μm. **e** Immunoblotting with anti-GST antibodies was used to detect the binding of recombinant His-ANT2 to GST-GRP75. **f** Coimmunoprecipitation of ANT2 and truncated domains of GRP75 (bottom panel). Flag-tagged wild-type GRP75 (GRP75-FL) or truncated GRP75 (GRP75-N and GRP75-C) were transfected into HEK293T cells. **g** Immunoblots (left panel) and real-time qPCR analysis (right panel) of ANT2 and GRP75 in differentiated 3T3-L1 adipocytes transfected with control vector (NC) or Myc-GRP75 plasmids. **h** Evaluation of the ANT2 half-life in GRP75-overexpressing adipocytes treated with cycloheximide. The indicated proteins were analysed by immunoblotting, and the quantification of ANT2 protein relative to the control β-actin by ImageJ software is shown at the bottom. **i** Ubiquitination assays of exogenous ANT2 in lysates from differentiated 3T3-L1 adipocytes transfected with NC or GRP75 plasmids. The data are presented as the mean ± SEM. The exact *P* values were tested with an unpaired two-tailed Student’s *t* test in (**g**)

To elucidate the mechanism of GRP75-mediated adipocyte browning, coimmunoprecipitation and mass spectrometry analyses of iWAT lysates from YES2 tumour-bearing mice were performed to identify endogenous GRP75-interacting proteins (Fig. 4a and Supplementary Table 3). Adenine nucleotide transporter 2 (ANT2), an inner mitochondrial membrane protein, not only functions as a classical ADP and ATP transporter but also plays a role in fatty acid-induced mitochondrial proton leakage.^32^ The fine-tuning of ANT2 in the balance between ATP generation and thermogenesis^32^ and its involvement in uncoupled respiration of normal BAT^33^ suggest that ANT2 may be involved in GRP75-induced adipocyte browning. Coimmunoprecipitation further confirmed that GRP75 bound to ANT2 in the iWAT of YES2 tumour-bearing mice and LLC tumour-bearing mice (Fig. 4b and Supplementary Fig. 6a). Consistently, a GRP75–ANT2 interaction was detected in adipocytes cotransfected with Flag-ANT2 and Myc-GRP75 plasmids (Fig. 4c). The results of the proximity ligation assay (PLA) demonstrated that adipocytes exhibited greater PLA signals following treatment with GRP75-overexpressing EVs than after treatment with the corresponding controls (Fig. 4d). Moreover, a glutathione S-transferase (GST) pull-down assay was performed by mixing recombinant GST-GRP75 and His-ANT2 proteins with anti-GST magnetic beads (Fig. 4e). Immunoblotting analyses of precipitated GST-GRP75 with a His-tag indicated that GRP75 directly interacted with ANT2. To identify the functional region of GRP75 that binds to ANT2, wild-type and two truncated GRP75 mutants were generated and co-transfected with ANT2 plasmids into HEK293T cells, respectively. Structural truncation analysis revealed that the interaction with ANT2 required the C-terminus (433–679 aa) of GRP75 (Fig. 4f), which contains a peptide-binding domain, indicating that GRP75 interacts with ANT2 in adipocytes via its peptide-binding structural domain.

To explore the effect of GRP75–ANT2 complex formation on the expression of ANT2, the protein level of ANT2 in differentiated 3T3-L1 adipocytes transfected with Myc-GRP75 plasmids was examined. Compared with those in control adipocytes, ANT2 protein levels were 3.9-fold greater in GRP75-overexpressing adipocytes, whereas ANT2 mRNA levels remained unchanged (Fig. 4g), indicating that GRP75 regulates ANT2 expression at the posttranslational level. Subsequently, a cycloheximide pulse-chase assay was performed to investigate whether GRP75 regulates the stability of ANT2 in adipocytes. As shown in Fig. 4h, the half-life of ANT2 was prolonged by GRP75 overexpression, indicating that GRP75 substantially inhibited the degradation of ANT2. To explore whether GRP75 regulates ANT2 stability via the ubiquitin‒proteasome pathway, an in vitro ubiquitination assay was carried out, and the results indicated that the overexpression of GRP75 decreased the ubiquitination of ANT2 in adipocytes (Fig. 4i). Taken together, these findings prove that ANT2 interacts with the peptide-binding domain of GRP75, which subsequently promotes the stability of ANT2 by decreasing its ubiquitination.

### ANT2–GRP75 promotes adipocyte browning by enhancing the interaction with UCP1 and its stability

**Fig. 5:**
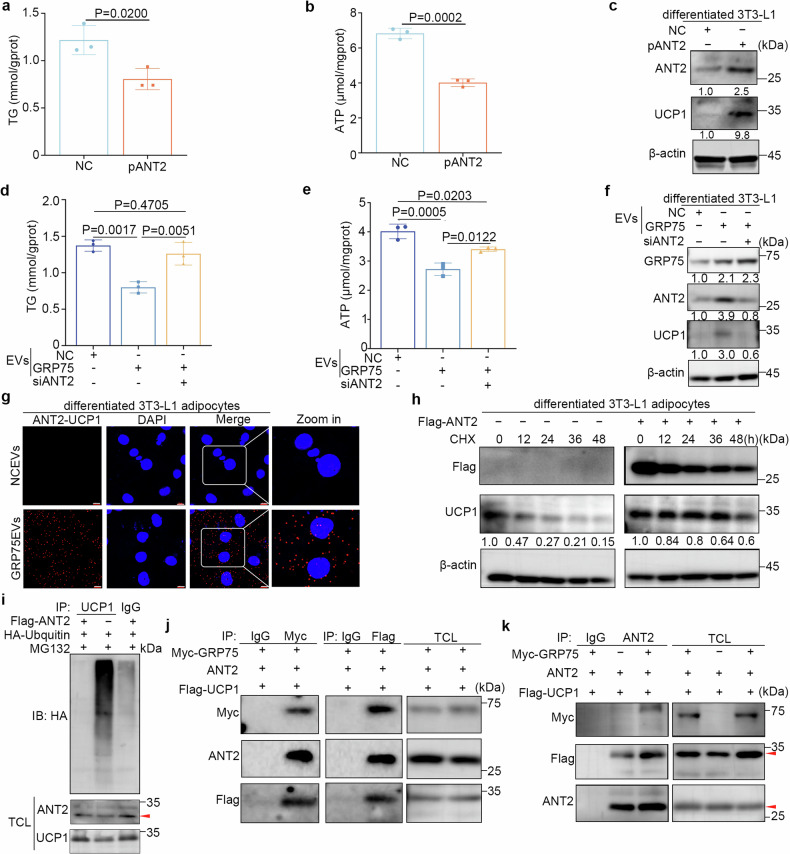
ANT2–GRP75 promotes adipocyte browning by enhancing the interaction with UCP1 and its stability. **a**, **b** Quantitation of intracellular TG (**a**) and ATP (**b**) levels in primary adipocytes transfected with control vector (NC) or Flag-ANT2 plasmids (pANT2). **c** Immunoblots showing ANT2 and UCP1 expression in differentiated 3T3-L1 adipocytes treated as described in **a**. **d**, **e** Quantitation of intracellular TG (**d**) and ATP (**e**) levels in primary adipocytes treated with NC-EVs or GRP75-EVs and transfected with si-NC or si-ANT2. **f** Immunoblots showing GRP75, ANT2, and UCP1 expression in differentiated 3T3-L1 adipocytes treated as described in **d**. **g** Proximity ligation assay signals and enlarged images of the combination of anti-ANT2 and anti-UCP1 in differentiated 3T3-L1 adipocytes treated with NC-EVs or GRP75-overexpressing EVs. DAPI, nuclei. Scale bar, 10 μm. **h** Immunoblots of UCP1 in differentiated 3T3-L1 adipocytes transfected with NC or Flag-ANT2 plasmids in the presence of cycloheximide. **i** Ubiquitination assays of UCP1 in lysates from differentiated 3T3-L1 adipocytes transfected with NC or Flag-ANT2 plasmids. **j** Coimmunoprecipitation of GRP75, ANT2, and UCP1. HEK293T cells were co-transfected with Myc-GRP75, ANT2, and Flag-UCP1. Protein extracts were immunoprecipitated with antibodies against Myc-tag or Flag-tag, followed by immunoblotting with the indicated antibodies. **k** Exogenous GRP75 enhances the ANT2–UCP1 interaction. Coimmunoprecipitation assays were performed against ANT2 using HEK293T cell lysates with or without transfection of Myc-GRP75. The quantification of protein relative to the control protein β-actin by ImageJ are shown at the bottom in (**c**, **f** and **h**). The data are presented as the mean ± SEM. The exact *P* values were tested with unpaired two-tailed Student’s *t* tests in (**a** and **b**) and one-way ANOVA in (**d** and **e**)

To examine whether the increase in ANT2 could induce adipocyte browning, ANT2-overexpressing adipocytes were generated by transfection with Flag-ANT2 plasmids, which caused a marked decrease in the intracellular TG and ATP levels (Fig. 5a, b). Immunoblotting indicated that UCP1 protein levels were 9.8-fold greater in ANT2-overexpressing adipocytes than in control cells (Fig. 5c), suggesting that ANT2 could play a positive role in regulating adipocyte browning. To determine the role of ANT2 in GRP75-mediated adipocyte browning, adipocytes transfected with si-ANT2 were subsequently coincubated with GRP75-overexpressing KYSE150-EVs. The results showed that ANT2 downregulation hindered the GRP75-induced decrease in intracellular TG and ATP contents in adipocytes (Fig. 5d, e). Correspondingly, the upregulation of UCP1 in adipocytes induced by GRP75-overexpressing EVs was suppressed by ANT2 knockdown (Fig. 5f), indicating that ANT2 is required for adipocyte browning caused by ectopic GRP75 expression.

Next, we elucidated the mechanisms underlying ANT2-induced adipocyte browning. The ANT family was found to interact with the subunits of various electron transport chain complexes to perform their functions.^34,35^ Interestingly, ANT2 expression was greater in BAT than in subcutaneous and visceral WAT of non-tumour-bearing mice, as previously described,^33^ and the interaction of ANT2 with UCP1 was determined using coimmunoprecipitation experiments followed by mass spectrometry (Supplementary Fig. 7a, b). These observations suggested that ANT2 might be an essential interacting protein of functional UCP1. To verify this hypothesis, we initially validated the interaction between ANT2 and UCP1, and the results revealed greater PLA signals for ANT2–UCP1 in adipocytes treated with GRP75-overexpressing EVs than in those treated with control EVs (Fig. 5g), indicating an increase in the interaction between UCP1 and ANT2. Immunoblot analysis of cells treated with cycloheximide revealed that ANT2 overexpression in adipocytes prolonged the half-life of UCP1 (Fig. 5h). Furthermore, the in vitro ubiquitination assay confirmed that the ubiquitination level of UCP1 was reduced upon ANT2 overexpression (Fig. 5i). These findings demonstrate that ANT2 binds to and stabilizes the UCP1 protein by decreasing its ubiquitination level, thereby inducing adipocyte browning.

To determine whether GRP75, ANT2, and UCP1 could form a complex, computational protein docking analysis was conducted to model the most energetically favoured pose of GRP75–ANT2–UCP1 in the potential binding sites, which suggested that the three proteins could assemble a stable complex (Supplementary Fig. 7c). Additionally, the lysates of HEK293T cells co-expressing Myc-GRP75, ANT2, and Flag-UCP1 were subjected to immunoprecipitation with anti-Myc and anti-Flag antibodies, respectively. Immunoblotting analysis indicated the formation of the GRP75–ANT2–UCP1 complex (Fig. 5j). Moreover, a two-step coimmunoprecipitation assay involving the immunoprecipitation of UCP1 with anti-Flag followed by a second round of immunoprecipitation with anti-ANT2 antibodies was conducted using the same lysates. The presence of all three components in the final immunoprecipitation data suggested the formation of a complex involving GRP75, ANT2, and UCP1 (Supplementary Fig. 7d). Subsequently, the potential role of GRP75 in facilitating the interaction between ANT2 and UCP1 was investigated by overexpressing GRP75 in HEK293T cells. Increased GRP75 expression promoted the ANT2–UCP1 interaction (Fig. 5k). These results demonstrate that GRP75 forms a complex with ANT2 and UCP1, facilitating their interactions and thereby enhancing UCP1 accumulation.

### GRP75 inhibitors alleviate adipocyte browning in vitro and in vivo

**Fig. 6:**
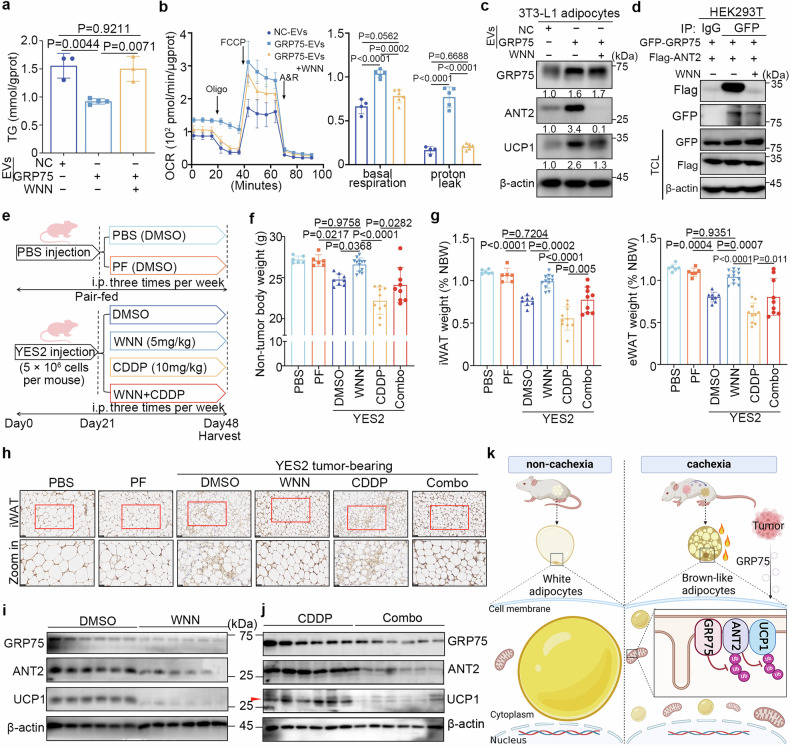
GRP75 inhibitors alleviate adipocyte browning in vitro and in vivo. **a** Quantitation of intracellular TG content in differentiated 3T3-L1 adipocytes treated with NC-EVs or GRP75-EVs in the presence or absence of the GRP75 inhibitor *withanone* (WNN). **b** OCR of differentiated adipocytes as described in **a**. Left: plot of the time course OCR normalized to the protein concentration. Right: calculated respiration levels of basal and proton-leaked OCRs. **c** Immunoblots of GRP75, ANT2, and UCP1 in differentiated adipocytes, as shown in **a**. **d** Coimmunoprecipitation of GRP75 and ANT2 in HEK293T cells treated with or without WNN. HEK293T cells were transfected with GFP-GRP75 plasmids and Flag-ANT2 plasmids. **e** Experimental design of the in vivo experiment. YES2 tumour-bearing mice were intraperitoneally injected with DMSO (DMSO), WNN (5 mg/kg), cisplatin (10 mg/kg, CDDP), or a combination of WNN with cisplatin (Combo). The PBS and PF groups included tumour-free mice injected with vehicle. **f** NBWs of the six groups described in **e** on day 48. **g** Ratios of iWAT (left) and eWAT (right) to NBW in **e** on day 48. **h** Representative IHC images of UCP1 in the iWAT from six groups. Scale bar, 50 μm. Magnified images labelled with a red rectangle are shown in the bottom panel. Scale bar, 25 μm. **i**–**j** Immunoblots of GRP75, ANT2, and UCP1 in the iWAT of the DMSO and WNN groups (**i**) or the CDDP and Combo groups (**j**) (*n* = 6; representative of six biological replicates per group). **k** Working model for how tumour extracellular vesicular GRP75 regulates white adipocyte browning in cachexia progression (created by Biorender.com). The data are presented as the mean ± SEM. *n* = 6 (PBS and PF), *n* = 8 (DMSO), *n* = 12 (WNN), *n* = 10 (CDDP) and *n* = 9 (Combo). The exact *P* values were tested with one-way ANOVA in (**a**, **b**, **f**, and **g**)

To test whether GRP75 could be considered a therapeutic target for treating WAT browning and atrophy, the effects of *withanone* (WNN), a GRP75 inhibitor,^36^ on adipocyte browning were examined. Notably, treatment with WNN hindered the reduction in intracellular TG (Fig. 6a) and the increase in basal and proton-leaked OCRs (Fig. 6b) induced by coincubation with GRP75-overexpressing EVs. In addition, WNN treatment prevented the upregulation of UCP1 expression in adipocytes (Fig. 6c). Coimmunoprecipitation analysis demonstrated that WNN effectively targeted the interaction between GRP75 and ANT2 (Fig. 6d). Taken together, these results demonstrate that WNN counteracts GRP75 overexpression-induced adipocyte browning in vitro.

To examine the effectiveness of WNN in inducing WAT browning and alleviating cachexia in vivo, DMSO, WNN, cisplatin (CDDP), or a combination of WNN and CDDP was administered intraperitoneally to YES2 tumour-bearing mice three times per week for four weeks (Fig. 6e). This intervention was initiated at the T-1 timepoint, coinciding with the observation of white adipose tissue atrophy and elevated expression of GRP75 and UCP1 in YES2 tumour-bearing mice (Fig. 6e). Tumour-free mice (PBS and PF) were used as negative controls (Fig. 6e). The WNN-treated mice showed substantial gains in NBW, whereas the DMSO-treated group displayed cachexia (Fig. 6f). WAT atrophy and skeletal muscle wasting in YES2 tumour-bearing mice were substantially suppressed by WNN (Fig. 6g and Supplementary Fig. 8a). Histological examination revealed that WNN treatment prevented a decrease in adipocyte size in the iWAT of tumour-bearing mice (Supplementary Fig. 8b), indicating the anti-cachexia activity of WNN. Furthermore, decreased UCP1 expression was observed in the iWAT of WNN-treated mice by immunohistochemistry and immunoblotting (Fig. 6h–i). Cisplatin is a widely used first-line treatment for a range of solid tumours and frequently promotes a cachexia-like state in patients.^3^ Notably, WNN-treated mice exhibited marked inhibition of tumour growth, comparable to that of CDDP-treated mice (Supplementary Fig. 8c–f). WNN treatment effectively ameliorated WAT browning and the subsequent cachectic state. Collectively, these findings demonstrate that WNN exerts marked anti-cachexia effects and suppresses ESCC growth in vivo.

The combination of WNN with CDDP substantially alleviated CDDP-induced NBW and WAT and skeletal muscle loss (Fig. 6f–g and Supplementary Fig. 8a), with significantly reduced WAT browning (Fig. 6h, j). In addition, biochemical parameters indicated that WNN treatment did not cause systemic toxicity in the major organs of the mice (Supplementary Fig. 9). These data indicate that WNN ameliorates cisplatin-induced fat browning without causing detectable toxicity.

Taken together, both in vitro and in vivo results suggest that GRP75 is a potential therapeutic target, and its inhibitor, WNN, might be a promising drug candidate for treating ESCC-associated cachexia.

## Discussion

In this study, we developed mouse models of progressive cancer cachexia that mimic clinical cachexia phenotypes and highlighted that WAT atrophy by browning is an important change that precedes skeletal muscle wasting during early cachexia development. By analysing differentially expressed proteins from cachexia-inducing tumour-derived EVs, GRP75 was identified as an essential regulator of tumour-induced WAT browning. Overexpression of GRP75 in the non-cachexia inducing cell line KYSE150 resulted in weight loss and WAT browning in vivo. Through verification and functional experiments on WAT from tumour-bearing mice and cell lines, we demonstrated that the formation of the GRP75–ANT2–UCP1 complex promoted the stability of UCP1 in GRP75-triggered WAT browning (Fig. 6k). Finally, we revealed that *withanone*, a GRP75 inhibitor, can alleviate WAT browning and weight loss in tumour-bearing mice. These findings demonstrate that tumour-derived GRP75 plays a vital role in mediating WAT browning during the progression of cancer cachexia.

To date, effective treatments for cancer cachexia are limited, partially because a considerable amount of research has focused on advanced or refractory cachexia. These patients often present with highly complex states, encompassing aspects such as tumour metastasis and drug resistance, accompanied by significant heterogeneity. Hence, we propose that exploring the features and underlying molecular mechanisms of early cachexia development may provide new insights into interventional treatments. Here, by setting three time points to detect cachexia progression in a mouse model of ESCC, we investigated the dynamic changes in physical parameters associated with cancer cachexia, including body weight, food consumption, adipose and skeletal muscle tissues, and serum inflammatory factors (Fig. 1 and Supplementary Fig. 1). At least 5% body weight loss, including atrophy of muscle and adipose tissues, as well as systemic inflammation, was observed in the YES2 tumour-bearing group at the endpoint of the current study (T-3) compared to its age- and sex-matched control group, which is in accordance with clinical cachexia phenotypes.^1^ The relatively slow growth of the ESCC cell line allowed the mouse model to better reflect the characteristics of physical alterations during the initiation and progression of cachexia, avoiding the short-term cachexia progression induced by aggressive and large tumours. These observations suggest that ESCC-bearing mice are a feasible model for studying the aetiology of cachexia and developing new therapeutic approaches.

Importantly, we detected the browning-associated markers UCP1 and PGC1α in a cachexia progression model and demonstrated that WAT atrophy by browning is an important and early manifestation of ESCC-induced cachexia development that occurs before skeletal muscle loss, consistent with previous observations in pancreatic adenocarcinoma.^15^ These studies, together with observations in LLC models (Supplementary Fig. 2), suggest that the involvement of WAT browning in the initiation and development of cachexia may not be limited to ESCC. In addition to murine models, WAT browning has also been detected in cachectic patients with cancer^15^ or other hypermetabolic diseases, such as burns,^37^ primary hyperparathyroidism,^38^ and chronic kidney disease.^39^ These studies emphasize the importance of maintaining functional WAT in preventing cachexia progression in a wide range of hypermetabolic disorders, indicating that the detection of adipose tissue content and properties may facilitate a comprehensive understanding of cachexia patients’ conditions.

As a member of the HSP70 family, GRP75 is highly expressed in thermogenic adipocytes;^30^ however, the specific mechanism of GRP75-mediated WAT browning in the context of cancer remains unclear. GRP75 was highly expressed in EVs from various cachexia-inducing tumour cells (Fig. 3d). By performing functional experiments using human- and murine-origin tumour EVs, GRP75 was demonstrated to be a crucial determinant of adipocyte browning in triggering cancer cachexia, suggesting that GRP75 may be essential for WAT browning in pan-cancerous cachexia. Notably, our findings from metabolic cage experiments, along with molecular analysis, clearly demonstrated that elevated GRP75 levels could trigger white adipose tissue browning and lead to weight loss (Fig. 3i–q). Additionally, a positive correlation was detected between increased GRP75 levels in the serum and progressive WAT atrophy in LLC tumour-bearing models (Supplementary Fig. 4h, R^2^ = 0.5234, *P* < 0.0001), suggesting the potential utility of GRP75 as a biomarker of WAT atrophy for monitoring cachexia progression.

Mechanistically, in vitro experiments with truncated proteins revealed that GRP75 was bound to and stabilized ANT2 by decreasing its ubiquitination. ANT2 has recently been described as having a fatty acid-induced H^+^ transporter function and is involved in mitochondrial oxidative respiratory chain uncoupling.^32^ Elevated protein expression of adenine nucleotide translocator was found in the WAT of cachectic patients,^40^ suggesting that ANT2 could be associated with cachectic adipose atrophy. Using computational protein docking and two-step coimmunoprecipitation, GRP75 was confirmed to form a complex with ANT2 and UCP1 to promote the accumulation of UCP1 (Supplementary Fig. 7c, d). Moreover, both the GRP75-induced reduction in intracellular TG and increase in uncoupled respiration were ameliorated in UCP1-knockdown adipocytes (Fig. 3f–h), suggesting that the GRP75-mediated browning of adipocytes is at least partially dependent on UCP1. Multiple studies have shown increased UCP1 expression in the WAT of cachectic mice and patients,^8,15,16^ and ablation of UCP1 could mitigate symptoms associated with cancer cachexia, including body weight loss and muscle wasting.^41^ This evidence indicates the importance of UCP1-induced increases in energy expenditure for cachexia-associated wasting. Furthermore, another brown adipose tissue marker, cell death-inducing DFFA-like effector A (CIDE-A), has been proposed to induce cachexia development independent of UCP-1, leading to excessive lipolysis via the inhibition of AMP-activated kinase (AMPK) in WAT.^14^ The differences in these findings may in part be due to the complexity of the pathogenic mechanisms involved in cachexia, as well as the large variety and heterogeneity of tumours.^9,42^ Notably, both UCP1-dependent and UCP1-independent increases in energy expenditure could occur in beige adipocytes.^43^ Therefore, the contribution of the UCP1-dependent pathway or other futile energy cycles to whole-body energy consumption in cachexia development under different pathological conditions or at different stages of disease progression remains to be assessed.

Previous clinical observations have demonstrated that a sequential loss of adipose tissue often precedes muscle wasting in patients with progressive cachexia,^8,10^ suggesting a specific order of events in cachexia progression. Despite recent advances in understanding the molecular basis of WAT atrophy in the context of cancer cachexia, little is known about adipose tissue-centred treatment regimens. This study revealed that WNN, a GRP75 inhibitor, prevented ESCC-induced adipocyte browning and considerably ameliorated cancer cachexia both in vitro and in vivo (Fig. 6). Although it is well documented that cytotoxic chemotherapy regimens, including cisplatin, can induce or exacerbate cachexia symptoms in cancer patients,^3^ to date, few approved treatments are available for counteracting their toxicity. Our results showed that in the absence of significant differences in the inhibitory effects on tumour growth between the WNN-treated and CDDP-treated groups, the WNN-treated mice exhibited increased body weight and ratios of adipose and muscle tissues (Fig. 6f, g and Supplementary Fig. 8a). In addition, WNN alleviated CDDP-induced cachexia in combination therapy without causing detectable toxicity (Supplementary Fig. 9). These findings suggest that WNN is a promising therapeutic option for treating ESCC-induced cachexia and mitigating the adverse effects of cisplatin.

The current study has several limitations. First, we did not distinguish between the subclasses of EVs that play a role in regulating adipocyte browning. Consistent with the recommendations of the International Society for Extracellular Vesicles, the term ‘extracellular vesicles’ was used for these vesicles.^20^ Second, the possibility that additional mediators secreted by tumours in addition to GRP75 might contribute to the phenotype of cachectic mice cannot be excluded. The lower expression of GRP75 in AsPC-1 and BxPC3 (human pancreas cancer) derived EVs indicated heterogeneity in the pathogenesis of cancer cachexia. The protection observed in adipocytes treated with GRP75-knockdown LLC-EVs was only partial, thus clarifying other mechanisms contributing to cachexia. Third, the long-term cytotoxicity of GRP75 inhibitors in vivo and in associated preclinical trials remains to be determined.

In summary, our findings suggest that alterations in both the content and function of adipose tissue play a pivotal role in early cachexia development, highlighting the critical importance of white adipose tissue remodelling in the occurrence and progression of cancer cachexia. The data shed light on the communication between tumour-derived GRP75 and adipocytes and the molecular mechanism by which GRP75 facilitates the formation of the ANT2–UCP1 complex to directly elicit adipocyte browning. These results expand the understanding of the processes leading to the development of cachexia and support the targeting of tumour-derived GRP75 as a promising therapeutic option for early cancer cachexia intervention.

## Materials and methods

### Antibodies and reagents

The following antibodies were purchased from Proteintech Group: anti-Alix (12422-1-AP), anti-CIDEA (13170-1-AP), anti-PGC1α (66369-1-Ig), anti-Flotillin-1 (15571-1-AP), anti-Lamin A/C (10298-1-AP), anti-HSP70 (66183-1-Ig), anti-TSG101 (28283-1-AP), anti-GRP75 (14887-1-AP), and anti-UCP1 (23673-1-AP), anti-LAMP2 (27823-1-AP). The following antibodies were purchased from Cell Signalling Technology: anti-ACC (3676S), anti-ANT2 (14671S), anti-DYKDDDDK tag (14793S), anti-GFP (2955S), anti-GRP78 (3177S), anti-GST-tag (2624S), anti-His-tag (12698S), anti-HA-tag (2367S), anti-HSL (4107S), anti-p-HSL (Ser563) (4139S), anti-Myc-Tag (2278S), anti-UCP1 (14670S), anti-GAPDH (5174S), and anti-rabbit and mouse IgG (7074/7076). The anti-mouse GRP75 (sc-133137) and anti-UCP1 (sc-293418) antibodies were obtained from Santa Cruz Biotechnology.

### Mouse models

Both five-week-old male nude BALB/c nude mice and six-week-old male C57BL/6J mice were purchased from Beijing HFK Bio-Technology Co., Ltd. (Beijing, China) and randomly assigned to each group. Mice were cohoused with littermates and maintained on a 12-hour light and 12-hour dark cycle at 23 ± 2°C with access to standard chow (forage from Beijing HFK Bio-Technology Co., Ltd.) and water unless stated otherwise. Mice were acclimatized to their environment for one week before experimentation. In different experiments, 5 × 10^6^ YES2 cells, 1 × 10^6^ LLC cells or vehicle (1 × PBS) were injected subcutaneously into the right flanks of six-week-old and seven-week-old mice. Tumour size (mm^3^ = larger diameter × smaller diameter^2^/2), total body weight (g), and food intake (g) were measured every three days after tumour cell injection. For the paired feeding experiment, the tumour-free mice were paired fed as previously described,^14^ when the food intake of tumour-bearing mice was steadily reduced. According to the food intake per gram of body weight of tumour-bearing mice multiplied by the total weights of the non-tumour-bearing mice, food was allocated to the tumour-free mice to exclude the effect of food intake on body weight. This tumour-free mice group was named as the paired-feeding (PF) group. From the beginning of the paired feeding, there was no food left in the PF group during the test period. Three time points were used to detect the dynamic changes in multiple physical indicators in ESCC-bearing mice (T-1 to T-3, Fig. 1a): T1 and T2, referred to as the clinical pre-cachexia stage, were diagnosed based on the criterion of total body weight loss <5% accompanied by anorexia. In this study, the establishment of T-1 helps to capture the initial alterations of cachexia, even before significant weight loss; T-3, designated the clinical cachexia stage, signifies the period when tumour-bearing mice exhibit a total body weight loss exceeding 5% compared to that of the PF group, aligning with the clinical staging criteria for cachexia with a total body weight loss of ≥5%.^1^ The animal handling and related experimentation mentioned above complied with the national and institutional policies for the Animal Welfare Act and were approved by the Cancer Institute and Hospital, Chinese Academy of Medical Sciences, and Peking Union Medical College (Ethical code: EAEC 2018-17).

For dynamic observation of YES2 tumour-bearing mice from T-1 to T-3, five mice were included in each control group, namely, the PBS and PF groups, and ten mice were included in the YES2 group at each time point. For dynamic observation of LLC tumour-bearing mice, five mice were included in each control group, namely, the PBS and PF groups, and ten mice were included in the LLC group at each time point. Two of the LLC tumour-bearing mice died during the T-2 to T-3 assay period; therefore, eight mice in the LLC group died at T-3.

For GRP75 in xenograft studies, KYSE150 LV-Flag-GRP75 or KYSE150 LV-control cells (5 ×10^6^/100 μL) were subcutaneously implanted into the right flanks of six-week-old male BALB/c nude mice. Six BALB/c nude mice were used for each group, including the PBS, LV-Control and LV-Flag-GRP75 groups. Other tests followed the steps outlined previously.

For drug administration experiments, six-week-old male mice were subcutaneously inoculated with YES2 (5 ×10^6^ cells/100 μL) when the average tumour size reached approximately 60 mm^3^ for subsequent grouping and drug administration. Tumour-bearing mice were divided into four groups based on the principle that there were no statistically significant differences in tumour size or body weight: DMSO (2%), cisplatin (CDDP, 10 mg/kg), *withanone* (WNN, 5 mg/kg), or a combination of both (Combo). All mice were injected intraperitoneally three times a week for four weeks. The mice in the Combo group were injected with two drugs at least four hours apart. All mice were weighed before injection, and the volume of each injection was no more than 200 μL. WNN (20 mg, HY-129692, MedChemExpress, USA) was dissolved in 2 mL of DMSO as a storage solution and diluted with saline before use. CDDP (100 mg, Qilu Pharmaceutical Co., Ltd., Shandong, China) was dissolved in 10 mL of 0.9% physiological saline (solution). Six mice were included in each control group, including the PBS and PF groups, and eight YES2 tumour-bearing mice were included in the DMSO group. Twelve mice were included in each group that received drugs, namely, the WNN, CDDP and Combo groups. After four weeks of drug administration, mice that died within one hour after drug injection were excluded. The final groups were the WNN group (*n* = 12), CDDP group (*n* = 10), and Combo group (*n* = 9).

### Metabolic characterization

Two tumour-bearing groups were housed at 22 °C singly in metabolic cages (Columbus Instruments, CLAMS) for 12 h to adapt to the environment before the measurements according to the ref. ^41^ Oxygen consumption, carbon dioxide production, heat production, and activity were recorded over a 24-hour light-dark cycle.

### Immunohistochemical staining

For immunohistochemical staining, adipose or tumour tissue sections were first dewaxed and hydrated. Next, antigen retrieval with EDTA was performed after blocking endogenous peroxidase activity using a 3% hydrogen peroxide solution. Nonspecific signals were blocked with goat serum, and sections were incubated with rabbit anti-Ki-67 (1:200, ab15580, Abcam), rabbit anti-UCP1 (1:400) or rabbit anti-GRP75 (1:2500) secondary antibodies overnight at 4 °C. The sections were incubated with goat anti-rabbit IgG HRP-polymer at room temperature, stained with 3,3’-diaminobenzidine, counterstained with hematoxylin, and finally sealed with neutral gum. Images of the immunohistochemical sections were obtained with a scanner and displayed by Aperio ImageScope 12.3.3 (Leica Biosystems, Germany), in which at least two pathologists counted the positive cells.

For the IHC of the human ESCC tissue microarray (HEsoS180Su11, Shanghai Outdo Biotech Co. Ltd., China), the dilution ratio of rabbit anti-GRP75 was 1:4000. This microarray included a total of 108 human ESCC samples (one without a GRP75 value was not included in the statistical analysis), 63 of which were matched adjacent normal tissue samples. Supplementary Table 2 shows the clinicopathological characteristics of the patients (Ethical code: YB M-05-02). The IHC score was the product of the staining intensity score and positive area score. The intensity score was determined as follows: 0, negative; 1, weak; 2, moderate; and 3, strong. The frequency of positive cells was scored as follows: 0, less than 5%; 1, 5% to 25%; 2, 26% to 50%; 3, 51% to 75%; and 4, greater than 75%. In this microarray, the IHC scores of GRP75 ranged from 0 to 12. Tumour tissues were divided into two groups based on the median (median = 8). For statistical analysis, scores between 0 and 8 were defined as low expression, while scores >8 were defined as high expression.

### Enzyme-linked immunosorbent assay (ELISA)

Blood samples were collected and clotted for two hours at room temperature before being centrifuged at 1000 × *g* for 15 min at 4 °C. The supernatants (serum) were collected and diluted with reference dilutions at different magnifications. Brij98 (436240, Sigma‒Aldrich) was added as a ref. ^23^. Then, the analysis was carried out according to the manufacturer’s instructions. Mouse IL-6 (abs520004) and TNF-α (abs520010) ELISA kits were purchased from Absin Biotechnology Co., Ltd. (Shanghai, China); mouse GRP75 ELISA kits (NBP2-76446) were obtained from Novus Biologicals (USA); and human GRP75 ELISA kits (ELH-HSPA9-A) were obtained from Raybiotech, Inc. (USA).

### Protein analysis

For immunoprecipitation experiments, 30 µL of protein A/G agarose magnetic beads (HY-K0202, MedChemExpress, USA) were conjugated with antibodies and lysis buffer at 4 °C. Later, the protein samples were incubated with preconjugated beads overnight at 4 °C on a rotating wheel. The next day, the supernatants were completely discarded using a magnetic stand, and the protein-bound beads were washed ten times with lysis buffer. Proteins were eluted by adding loading buffer, heated at 100 °C for 10 min, and subjected to immunoblotting. ImageJ software was used to analyse the grey values of the immunoblotting protein bands.

Two-step coimmunoprecipitation was carried out as previously described^44^. First, the cell lysates were incubated with anti-Flag magnetic beads (HY-K0207, MedChemExpress) overnight, followed by elution with 1 mg/mL of the Flag polypeptide. A second round of immunoprecipitation was performed with the eluates containing anti-ANT2 or control IgG. Finally, the precipitated proteins were analysed by immunoblotting.

For the GST pull-down assay, recombinant His-ANT2 protein (Ag11956, Proteintech Group) was incubated with GST or recombinant GST-GRP75 protein (Ag6673, Proteintech Group) and anti-GST magnetic beads (HY-K0222, MedChemExpress) at 4 °C overnight. The expression of the GST tag and His tag was confirmed by immunoblotting.

### Cell culture and transfection

ESCC cell lines were a gift from Yoshikazu Shimada of Kyoto University (Sakyo-ku, Kyoto-shi, Kyoto, Japan). The cell lines 3T3-L1 (SCSP-5038), LLC (TCM 7), and HepG2 (SCSP-510) cells were purchased from the National Collection of Authenticated Cell Cultures (Shanghai, China). The AsPC-1, BxPC3, C26 and MC38 cell lines were provided by Peking Union Medical College Hospital (Beijing, China). The ESCC cell lines, AsPC-1 and BxPC3 were cultured in complete RPMI-1640 medium (03.4007 C; Eallbio). HEK293T, LLC, C26, MC38 and HepG2 cells were cultured in complete DMEM (03. PM1002D; Eallbio). The cell culture conditions were maintained at 37 °C with 5% CO_2_. When 3T3-L1 cells reached 100% confluence, they were induced with a cocktail containing 1 μM dexamethasone (Sigma‒Aldrich, D4902), 10 μg/ml insulin, and 0.5 mM isobutylmethylxanthine (Sigma, I7018) for four days and then cultured for another 2 days in DMEM containing 10 μg/ml insulin. Finally, the cells were cultured in DMEM supplemented with 10% FBS for two days, at which time the induction efficiency reached more than 90%.

For coculture experiments, differentiated 3T3-L1 cells were seeded in the bottom chambers of Transwell plates. YES2, KYSE150, LLC, and SHEE (1 × 10^5^/well) were added to the upper compartments. Differentiated 3T3-L1 cells were collected after 48 h of coincubation.

For differentiation of the stromal vascular fraction (SVF) into adipocytes, preadipocytes were isolated from the inguinal WAT of three-week-old C57BL/6J male mice. The WAT was minced and placed in a constant temperature shaker at 37 °C for half an hour of digestion in type II collagenase. The digestion was terminated by the addition of an equal volume of complete DMEM. The suspension was centrifuged at 400 rpm for five minutes to remove the supernatant. The cells were resuspended and filtered through a 40-μm cell strainer and then inoculated and cultured in culture dishes containing complete DMEM. The procedures used to differentiate the SVFs were the same as those described previously.^9^

The mouse/human Myc-GRP75, GFP-GRP75, Flag-GRP75 (full length), Flag-GRP75 (1–432 aa), Flag-GRP75 (433–679 aa), and Myc-ANT2 plasmids were constructed by Beijing Maijin Biotechnology Co., Ltd. (Beijing, China). Both specific siANT2 and siUCP1 sequences were purchased from RiboBio Co., Ltd. (Guangzhou, China) and are as follows: si-ANT2-1, 5′-CCAATGTCATCAGATACTT-3′; si-ANT2-2, 5′-CCTGGTTAAGATCTACAAA-3′; and si-UCP1, 5′-CCATCTGCATGGGATCAAA-3′. Differentiated adipocytes were transfected with siRNAs or plasmids using Lipofectamine 2000 (Thermo Fisher) according to the manufacturer’s instructions. For stable transfection of GRP75 short hairpin RNA (shRNA), YES2 cells were transfected with pLenti‐hU6-GRP75-shRNA-CBh-gcGFP-IRES-Puro. Control cells were transfected with pLenti‐hU6‐NC‐shRNA‐CBh-gcGFP-IRES-Puro. For stable transfection of the GRP75-overexpressing plasmid, pCMV-Ubi-GRP75-3Flag-CBh-gcGFP-IRES-Puro was transfected into KYSE150 cells. Briefly, the virus solution was added to the complete medium, and the cells were infected with 10 μg/mL polybrene. Cells transfected with pCMV-Ubi-3FLAG-CBh-gcGFP-IRES-Puro were used as negative controls. All viruses were purchased from Shanghai GeneChem Co., Ltd. The constructed cell lines were cultured in complete medium supplemented with 0.5 μg/mL puromycin (S7417, Selleck, Houston, USA).

### Extracellular vesicle isolation, quantification and characterization

All steps below followed the guidelines of MISEV2023.^20^ Cells were cultured to 80–90% confluency in 100 mm culture dishes, and the medium was replaced with serum-free media (containing 1% penicillin/streptomycin) for 48 h. EVs were harvested by differential ultracentrifugation as described in the literature.^25^ The conditioned media were collected and centrifuged continuously at 4 °C as follows: 300 × *g* for 10 min, 2000 × *g* for 10 min, 10,000 × *g* for 30 min, and 100,000 × *g* for 70 min. After centrifugation at 300 × *g*, the supernatant was filtered through a 0.22 μm filter before proceeding with the next steps. The pellets (EVs) were suspended in an appropriate volume of PBS for subsequent experiments.

EVs were examined via transmission electron microscopy (TEM-1400 Plus) at 80 kV as described previously.^24^ The concentration of tumour-derived EVs added to adipocytes was estimated by NanoSight to be approximately 1 × 10^11^/mL. To inhibit EV release, YES2 cells were incubated with serum-free media supplemented with 10 μM GW4869 (S7609, Selleck) for 24 h, and YES2-EVs were isolated after treatment.

### Mass spectrometry (MS) analysis

MS experiments were performed on a Q Exactive HF-X mass spectrometer that was coupled to an Easy nLC 1200 (Thermo Scientific, Scotts Valley, CA, USA). For proteins differentially expressed between YES2-EVs and KYSE150-EVs, MS data were analysed using MaxQuant software version 2.0.1.0 and were searched against the UniProtKB Human database (204995 total entries; https://www.uniprot.org/UniProt/?query=taxonomy:9606; downloaded 06/24/2022). For proteins interacting with GRP75, MS data were analysed using MaxQuant software version 1.5.8.3 and were searched against the UniProtKB Mouse database (88108 total entries; downloaded 11/05/2021). The database search results were filtered and exported with a < 1% false discovery rate (FDR) at the peptide and protein levels. This part of the work was supported by Shanghai Bioprofile Technology Company Ltd.

### Measurement of the oxygen consumption rate

The cellular oxygen consumption rate (OCR) of differentiated 3T3-L1 cells under different treatments was measured using an Agilent Seahorse XFe96 (Agilent Technologies Co. Ltd., USA) and analysed by Agilent Seahorse Wave Software. 3T3-L1 cells (5 × 10^3^ cells/well) were seeded in 0.2% gelatin-coated microplates and differentiated as described above. Before testing, the medium was changed to Agilent Seahorse XF Assay Medium, which included 25 mM glucose and 1 mM pyruvate, in a non-CO_2_ incubator for one hour. The oxygen consumption rate of adipocytes was determined using 2 μM oligomycin, 1 μM FCCP, and 10 μM antimycin A and rotenone. The OCRs were normalized to the protein concentrations.

### Immunofluorescence

For the internalization of EVs, EVs were labelled with PKH67 using PKH67 Fluorescent Cell Linker Kits (P7333, Sigma‒Aldrich) according to the manufacturer’s protocols. Differentiated adipocytes were cultured in Nunc glass bottom dishes and treated with PKH67-labelled EVs for eight hours in cell incubators. Then, the cells were fixed with 4% paraformaldehyde and treated with 0.2% Triton-X for 10 min before blocking. The primary anti-ACC was incubated with the cells at a dilution of 1:200 in the dark overnight at 4 °C. Then, the cells were stained with Alexa Fluor^®^ 594 secondary antibody (ZSGB-BIO) at a dilution of 1:200 at room temperature for one hour. The cells were stained with DAPI before observation, and the cells were sealed with 60% glycerol. Images of stained cells were acquired using a laser scanning confocal microscope (Leica Microsystems Heidelberg GmbH, Am Friedensplatz, Germany).

For colocalization, adipocytes were fixed with 4% PFA, permeabilized with 0.2% Triton X-100 and blocked with Duolink blocking buffer at 37 °C for one hour. Then, adipocytes were incubated with primary antibody (1:200) at 4 °C overnight. After washing, Duolink^®^ PLA experiments were performed according to the Duolink^®^ PLA Fluorescence experiment protocol (Merck). All cells were incubated with Duolink^®^ anti-rabbit PLUS (DUO92002, Merck) and anti-mouse MINUS PLA (DUO92004, Merck) probes, and PLA signals were generated using Duolink^®^ In Situ Detection Reagent (Far Red). Sections were blocked with Duolink^®^ In Situ Blocker (containing DAPI).

### Quantification and statistical analysis

All the statistical analyses were performed with at least three biological replicates. All the data are presented as the means ± standard errors (SEMs) unless otherwise stated in the figure legends. The data were subjected to normal distribution analysis before difference analysis by GraphPad Prism version 8.0. When the data conformed to a normal distribution, an unpaired two-tailed Student’s *t* test was used to compare the two groups. One-way analysis of variance (ANOVA) with Tukey’s post hoc test was used for comparisons among three or more groups. The statistical parameters used are described in the figure legends. Significance was set at *P* < 0.05, and the exact *P* values are labelled in the panels. The associations between GRP75 expression and clinicopathologic features were analysed by the chi-square (χ^2^) test. The Kaplan‒Meier method and log-rank test were used to assess the association between GRP75 expression and overall survival. *P* < 0.05 indicated statistical significance.

## Supplementary information

Supplementary_Materials

## Data Availability

All data and materials are presented in the main manuscript or supplementary materials, and can be obtained from the corresponding authors upon authorization or reasonable request.
