# Supplementary material for: GRP75 triggers white adipose tissue browning to promote cancer-associated cachexia
Source: Signal Transduct Target Ther. 2024 Sep 26;9:253. doi: 10.1038/s41392-024-01950-w (PMC11427701; doi:10.1038/s41392-024-01950-w)

Supplementary Materials for

**GRP75 triggers white adipose tissue browning to promote cancer-associated cachexia**

Xu Chen, Qingnan Wu, Wei Gong, Shaolong Ju, Jiawen Fan, Xiaohan Gao, Xingyang Liu, Xiao Lei, Siqi Liu, Xiangdong Ming, Qianyu Wang, Ming Fu, Yongmei Song, Yan Wang*, Qimin Zhan*

.

Correspondence to: Qimin Zhan (zhanqimin@bjmu.edu.cn) or Yan Wang (wy305@126.com)

**This PDF file includes:**

Materials and Methods

Supplementary Text

Figures. S1 to S9

Tables S1 to S3

Captions for Data S1 to S2

**Other Supplementary Materials for this manuscript include the following:**

Data S1 to S2

S1. Western blot bands

S2. Ethical Approval Form for Immunohistochemistry Chips in Human Esophageal Squamous Cell Carcinoma

Materials and Methods

**Methods**

**Hematoxylin and eosin staining**

Subcutaneous white adipose tissue (WAT) was obtained from the inguinal region (iWAT), visceral WAT was obtained from the peri-epididymal region (eWAT), and the gastrocnemius muscle (GA) was obtained from the calves of the mice. The analysis was performed as previously reported.^15^ Changes in the mean cross-sectional area relative to the number of adipocytes (A.U.) were assessed in the different groups.

**Protein analysis**

Protein samples were extracted from cells or frozen tissues with ice-cold lysis buffer (1% NP-40 with protease inhibitor cocktail) and centrifuged at 12,000 × g for 15 min. The supernatant was mixed with 5 × SDS loading buffer following heating. Protein extracts were separated by SDS‒PAGE and blotted onto polyvinylidene difluoride membranes (Bio-Rad, USA). The membranes were blocked with 5% skim milk and probed with primary antibodies and secondary HRP-conjugated antibodies. The signal intensity was detected by an Amersham Imager 600 (GE, Boston, MA, USA). β-actin was utilized as a reference housekeeping protein.

**Oil Red O staining**

Oil Red O powder (Sigma‒Aldrich) was dissolved in 100% isopropanol at 37°C for 12 h. The stock solution was diluted with water 3:2 for subsequent experiments. 3T3-L1 adipocytes were fixed in 4% paraformaldehyde for 30 min and moistened with 60% isopropanol for 40 s. The cells were stained with a working solution for 30 min and washed successively with isopropanol and ddH2O twice. Stained adipocytes were observed under a light microscope.

**Measurement of triglyceride and ATP contents**

Differentiated adipocytes were lysed with ice-cold 1% Triton-100 lysis buffer. Four microliters of cell lysate were aspirated and quantified using a triglyceride (TG) assay kit (A111-1-1, Nanjing Jiancheng Biological Product Co. Ltd., China). The intracellular TG contents of adipocytes were normalized to the corresponding protein concentrations.

An Enhanced ATP Assay Kit (Beyotime Biotechnology) was used to measure the ATP content of adipocytes subjected to different treatments according to the manufacturer’s protocol. The ATP content in the cells was normalized to the protein concentration.

**Silver staining**

Equal amounts of protein were subjected to electrophoresis. The gel was incubated with fixative solution overnight according to the instructions of the fast silver stain kit (P0017S, Beyotime Biotechnology, Shanghai, China). Briefly, the fixed solution was discarded, and 30% ethanol was added at room temperature for 10 min. Then, double distilled water was used to wash the gel for 15 min. The gel was sensitized for 2 min, and silver dye was added for 10 min. After the gel was washed, silver dye color developing solution was added until the ideal protein bands appeared. The stop solution was used to terminate the response. The ideal bands were cut for mass spectral analysis.

**Isolation of adipocyte mitochondria**

Primary adipocytes were treated with NC-EVs or GRP75-OE EVs for 48 hours. The mitochondria of adipocytes were isolated with a MinuteTM mitochondrial isolation kit (MP-007, Invent Biotechnologies, INC.), and the proteins of mitochondria from adipocytes were extracted with 1% NP-40, followed by SDS‒PAGE.

**RNA extraction and quantitative real-time polymerase chain reaction analysis**

Total RNA was isolated from cultured cells or frozen tissue samples using TRIzol reagent according to the manufacturer’s instructions and quantified using a NanoDrop One spectrophotometer (Thermo Fisher, 701-058112). cDNA was obtained by reverse transcription using the Prime ScriptTM RT reagent Kit with gDNA Eraser (Takara Biomedical Technology Co. Ltd., Beijing, China). cDNAs were subsequently amplified using the TB Green® Premix Ex Taq™ II (Tli RNaseH Plus) kit and a 7500 real-time PCR system (Applied Biosystems, Thermo Fisher, Waltham, MA, USA) according to the manufacturer’s protocols. RNA expression data were quantified using the ∆Ct method and normalized to the levels of housekeeping genes. Primers were designed according to Primer3 Input (version 0.4.0) and provided by Beijing Tianyi Huiyuan Co. Ltd. (Beijing, China). The primers used were as follows:

GRP75 forward primer 5’-TGGGATTGTGCACGTTTCTG-3’ and reverse primer 5’-TTCCTTCTTCCTGCGGTCT-3’;

ANT2 forward primer 5’-ACCTGGCCAATGTCATCAGA-3’ and reverse primer 5’-GAACTGGGTCCTCTTGTCCA-3’;

Atrogin-1 forward primer 5′-CAGCTTCGTGAGCGACCTC-3′ and reverse primer 5′-GGCAGTCGAGAAGTCCAGTC-3′;

MURF1 forward primer 5′-GTGTGAGGTGCCTACTTGCTC-3′ and reverse primer 5′-GCTCAGTCTTCTGTCCTTGGA-3′;

mtDNA forward primer 5’-CGAAAGGACAAGAGAAATAGAG-3’ and reverse primer 5’-GAACAAGGTTTTAAGTCTTACGCA-3’;

GAPDH forward primer 5′-CATGGCCTTCCGTGTTCCTA-3′ and reverse primer 5′-CCTGCTTCACCACCTTCTTG-3′;

18S RNA forward primer 5’-CAGCCACCCGAGATTGAGCA-3’ and reverse primer 5’-TAGTAGCGACGGGCGGTGT-3’;

The primers used were as follows: β-actin forward primer, 5’-ATGGAGGGGAATACAGCCCC-3’; reverse primer, 5’-TTCTTTGCAGCTCCTTCGTT-3’.

Supplementary Text


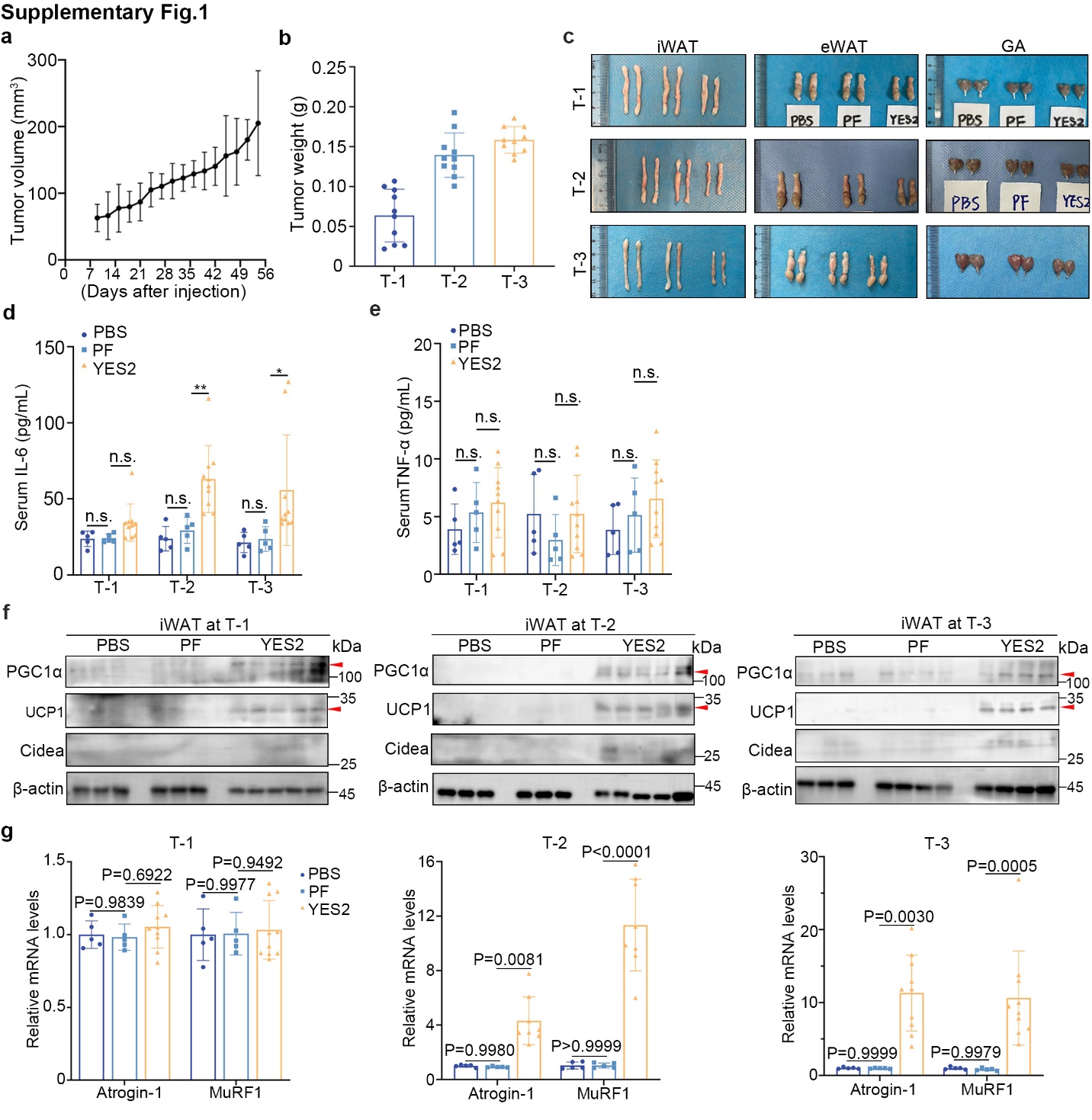


Figure. S1. WAT atrophy precedes skeletal muscle wasting during early cachexia development in YES2 tumor-bearing mice

**a** Subcutaneous tumor growth curve from YES2 tumors. **b** Weights of xenograft tumors of YES2 groups at three time points (n = 10). **c** Representative images of iWAT, eWAT, and GA from PBS, PF, and YES2 groups at three time points. **d, e** Serum levels of IL-6 (**e**) and TNF-α (**f**) in three groups at three time points. **f** Immunoblots of UCP1, PGC1α and CIDE-A in the iWAT from three groups at three time points. **g** Real-time PCR analysis of *Murf1* and *Atrogin1* in GA from three groups at three time points. The data are presented as mean ± SEM (n = 5 in each group, including PBS and PF groups, n =10 in YES2 group at each time point). The exact *P* values were tested with one-way ANOVA in (**d**, **e** and **g**). *: P < 0.05; **: P < 0.01; ***: P < 0.001; n.s.: no significance; iWAT: inguinal white adipose tissue; eWAT: epididymal WAT; iBAT: interscapular brown adipose tissue; GA: gastrocnemius.


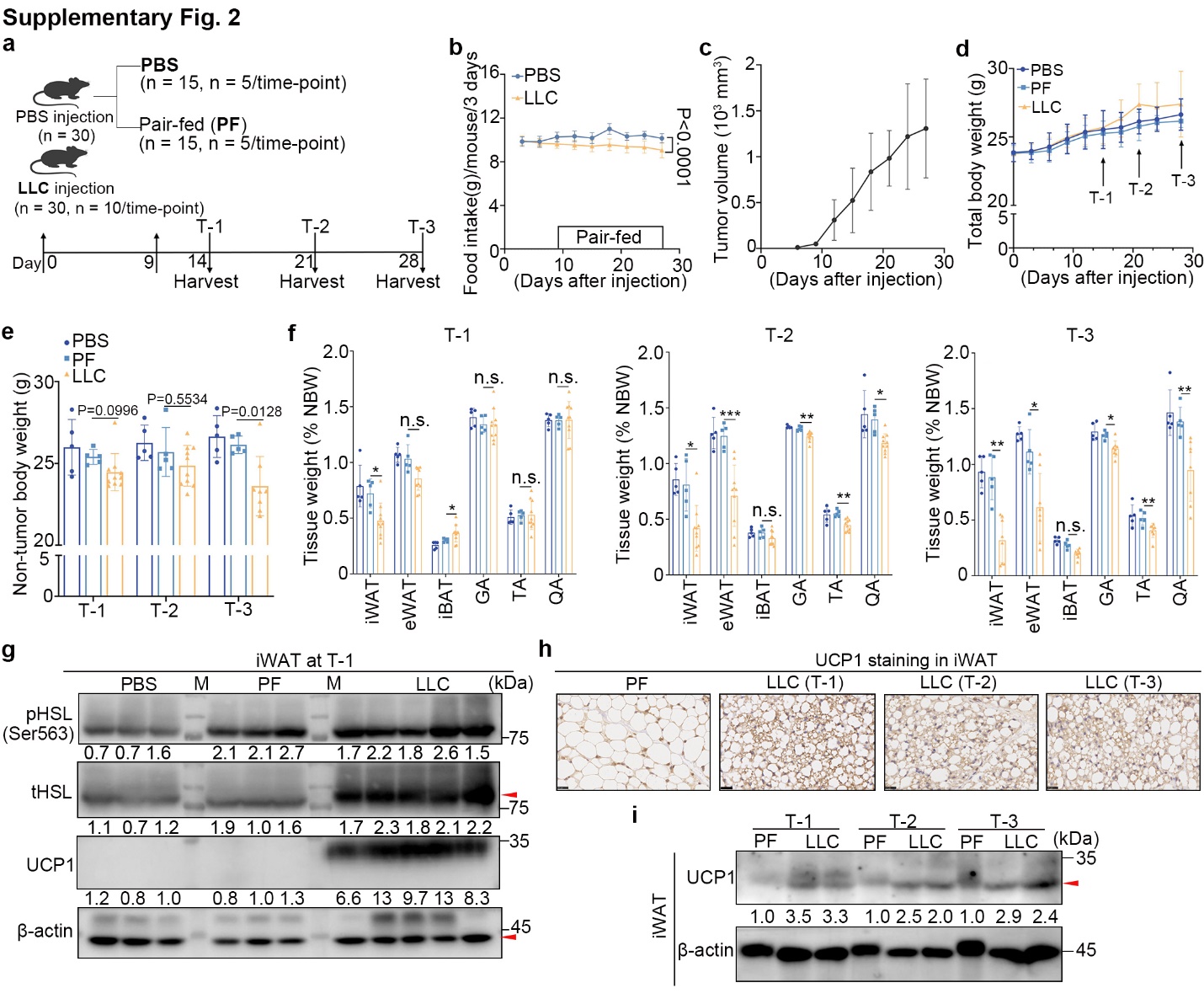


Figure. S2. The phenomenon that WAT atrophy precedes skeletal muscle loss is also observed in LLC-bearing mice

**a** Schematic diagram of *in vivo* subcutaneous injection of PBS or LLC cells in seven-week-old C57BL/6J mice and collected corresponding samples at three time points. The pair-fed (PF) group were set up on day 9 post-inoculation. **b** Food intake curves of PBS and LLC groups. **c** Subcutaneous tumor growth curve from LLC tumors. **d** Total body weight (TBW) curves of the PBS, PF, and LLC groups. **e** Non-tumor body weight (NBW) of three groups at three time points. **f** Ratios of adipose tissue (iWAT, eWAT, and iBAT) and skeletal muscle (GA, TA, and QA) to NBW in three groups at T-1, T-2, and T-3. **g** Immunoblots of p-HSL (Ser563), total HSL, and UCP1 in iWAT of three groups. **h** Representative immunohistochemistry (IHC) images of UCP1 in iWAT of LLC groups at three time points. The images of PF groups were shown as negative controls. Scale bar, 25 μm. **i** Immunoblots of UCP1 in iWAT of PF and LLC groups at three time points. The iWAT of PF mice at T-1was considered as a negative control. The data are presented as mean ± SEM (n = 5 mice in PBS and PF groups at each time point; n =10 mice in LLC groups at T-1 and T-2; n = 8 mice in LLC group at T-3). The exact *P* values were tested with unpaired two-tailed Student’s *t*-test in (**b**), multiplied *t*-test in (**c**), and one-way ANOVA in (**e, f**). *: P < 0.05; **: P < 0.01; ***: P < 0.001. n.s.: no significance;


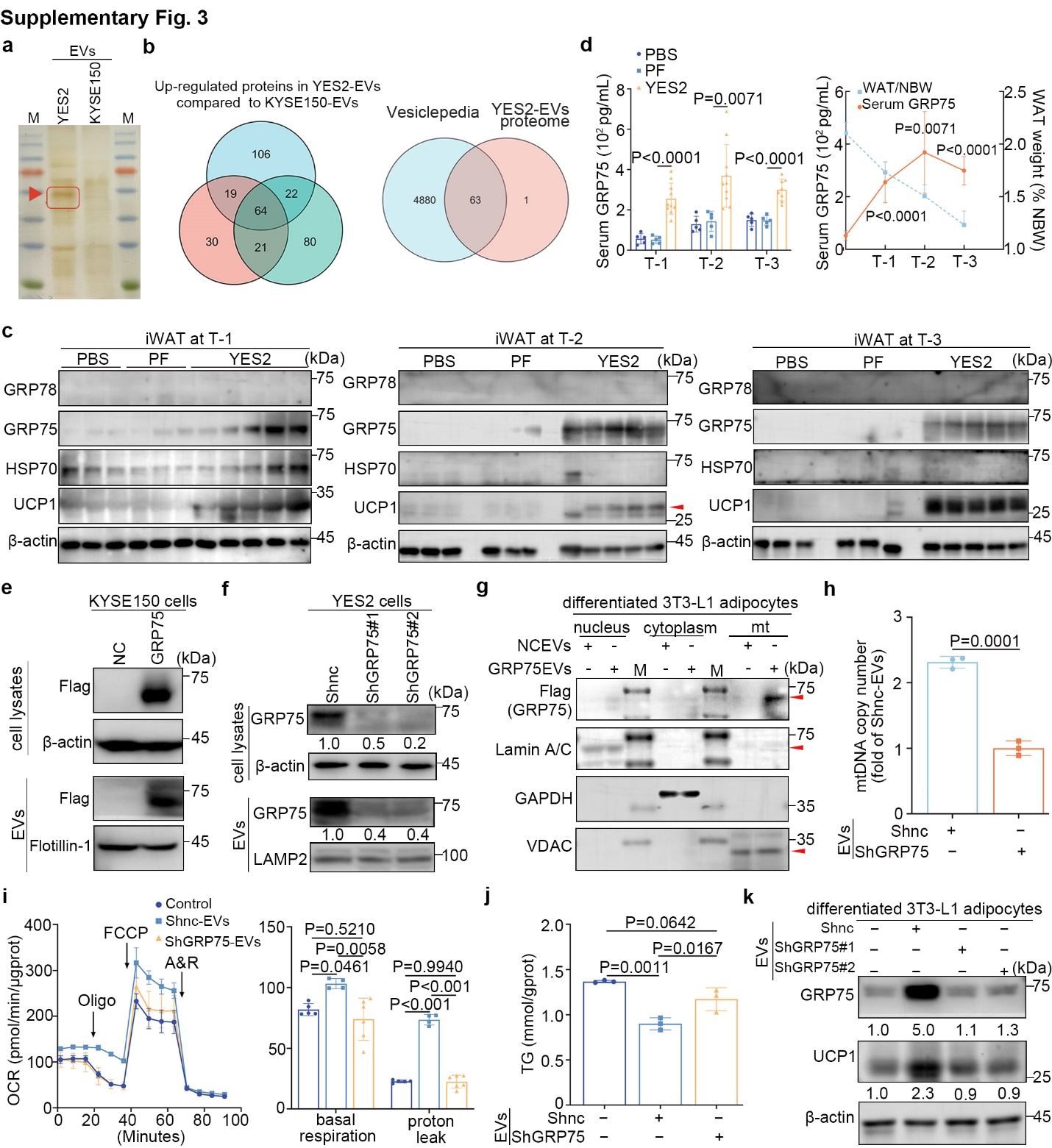


Figure. S3. GRP75 is essential for adipocyte browning

**a** Identification of highly-expressed proteins in YES2-EVs with respect to YES2-EVs and KYSE150-EVs lysates. The same amounts of proteins from two kinds of EVs were subjected to SDS-PAGE, followed by silver staining. The differential gel piece framed by the red rectangle was analyzed by mass spectrometry. EVs: extracellular vesicles; M: molecular mass markers. **b** Venn diagram of the 64 overlapping genes (unique peptides ≥ 4) from three independent experiments and Venn diagram of YES2-EVs proteome with 63 proteins reported in the Vesiclepedia database after filtering are shown on the right side. “Human” species for analysis. **c** Immunoblots of GRP78, GRP75, HSP70, and UCP1 expression in iWAT from PBS, PF, and YES2 groups at three time points. **d** Serum GRP75 levels of PBS, PF, and YES2 groups at three time-points (left) and correlation of WAT (iWAT and eWAT) ratios with serum GRP75 in YES2 groups (right). Ratios of WAT to NBW of tumor-free mice were considered baseline. Two outliers were excluded in the YES2 group at T-3. **e** Immunoblots of GRP75 in KYSE150 stably overexpressed GRP75 cells and corresponding EVs. **f** Immunoblots of GRP75 in the YES2 stably knockdown GRP75 cells and corresponding EVs. **g** Immunoblotting of Flag (GRP75), Lamin A/C, VDAC and GAPDH in the lysates of cytoplasm and mitochondria from the differentiated 3T3-L1 adipocytes treated with NC-EVs and GRP75-overexpressing EVs, respectively. mt, mitochondrion. **h** Evaluation of the mtDNA copy number in the cells treated with Shnc-EVs or ShGRP75-EVs. **i** Mitochondrial stress tests in the differentiated 3T3-L1 adipocytes treated with Shnc-EVs or ShGRP75-EVs. The cells treated with exo-free medium were shown as a negative control. Left: plot of time course OCR normalized by protein concentration. Right: calculated respiration levels of basal and proton-leak OCR. **j** Quantitation of intracellular triglyceride (TG) contents in the differentiated 3T3-L1 adipocytes as described in (**e**). **k** Immunoblots of GRP75 and UCP1 in the adipocytes as described in (**j**). The data are presented as means ± SEM. The exact P values were tested with unpaired two-tailed Student’s t-test in (**d**) and one-way ANOVA in (**e** and **f**). The data are presented as mean ± SEM. The exact *P* values were tested with one-way ANOVA in (**d**, **i** and **j**) unpaired two-tailed Student’s *t*-test in (**h**).


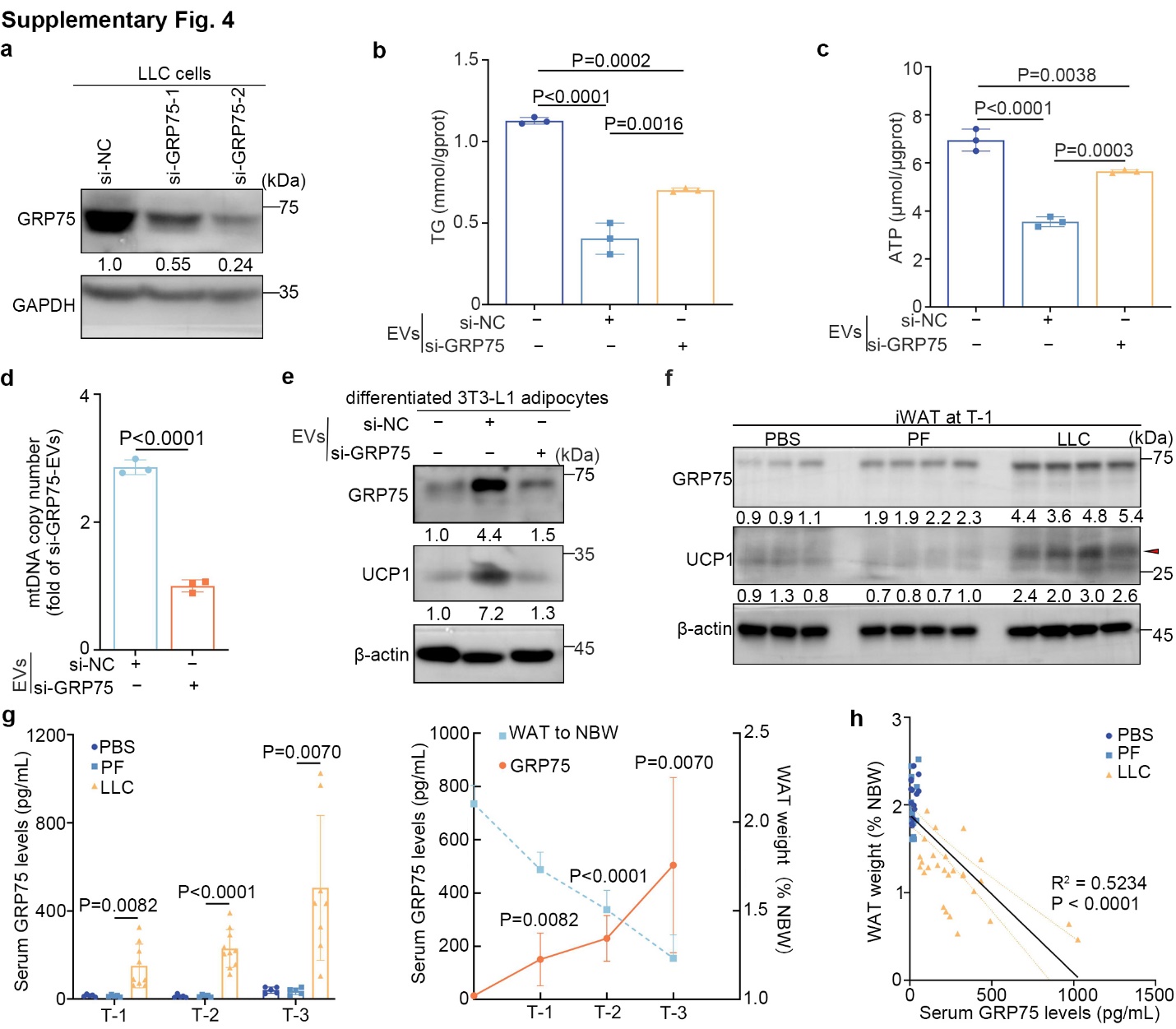


Figure. S4. GRP75 is essential for adipocyte browning

**a** Immunoblots of GRP75 in LLC transfected with si-NC or si-GRP75. **b**, **c** Quantitation of intracellular TG (**b**) and adenosine triphosphate (ATP) (**c**) contents in differentiated 3T3-L1 adipocytes treated with si-NC EVs or si-GRP75 EVs. The differentiated 3T3-L1 adipocytes treated with exo-free medium were considered as negative controls. **d** MtDNA copy number was detected in the differentiated 3T3-L1 adipocytes treated as described in (**b**). **e** Immunoblots of GRP75 and UCP1 in the adipocytes treated as described in (**b**). **f** Immunoblots of GRP75 and UCP1 in iWAT from PBS, PF, and LLC groups (n = 3–4; representative of 3–4 biological replicates per group). **g** Serum GRP75 concentration of three groups at three time points analyzed by ELISA and inverse correlation of WAT (iWAT and eWAT) ratios with serum GRP75 in LLC groups shown on the right side. WAT ratios of tumor-free mice were considered as baseline. One outlier in the LLC group at T-1 has been excluded. **h** Linear regression analysis comparing serum GRP75 concentration and ratios of WAT weight to NBW in three groups (R^2^ = 0.5234, *P* < 0.0001). The data are presented as mean ± SEM. The exact *P* values were tested with one-way ANOVA in (**b**, **c**, and **g**) and unpaired two-tailed Student’s *t*-test in (**d**).


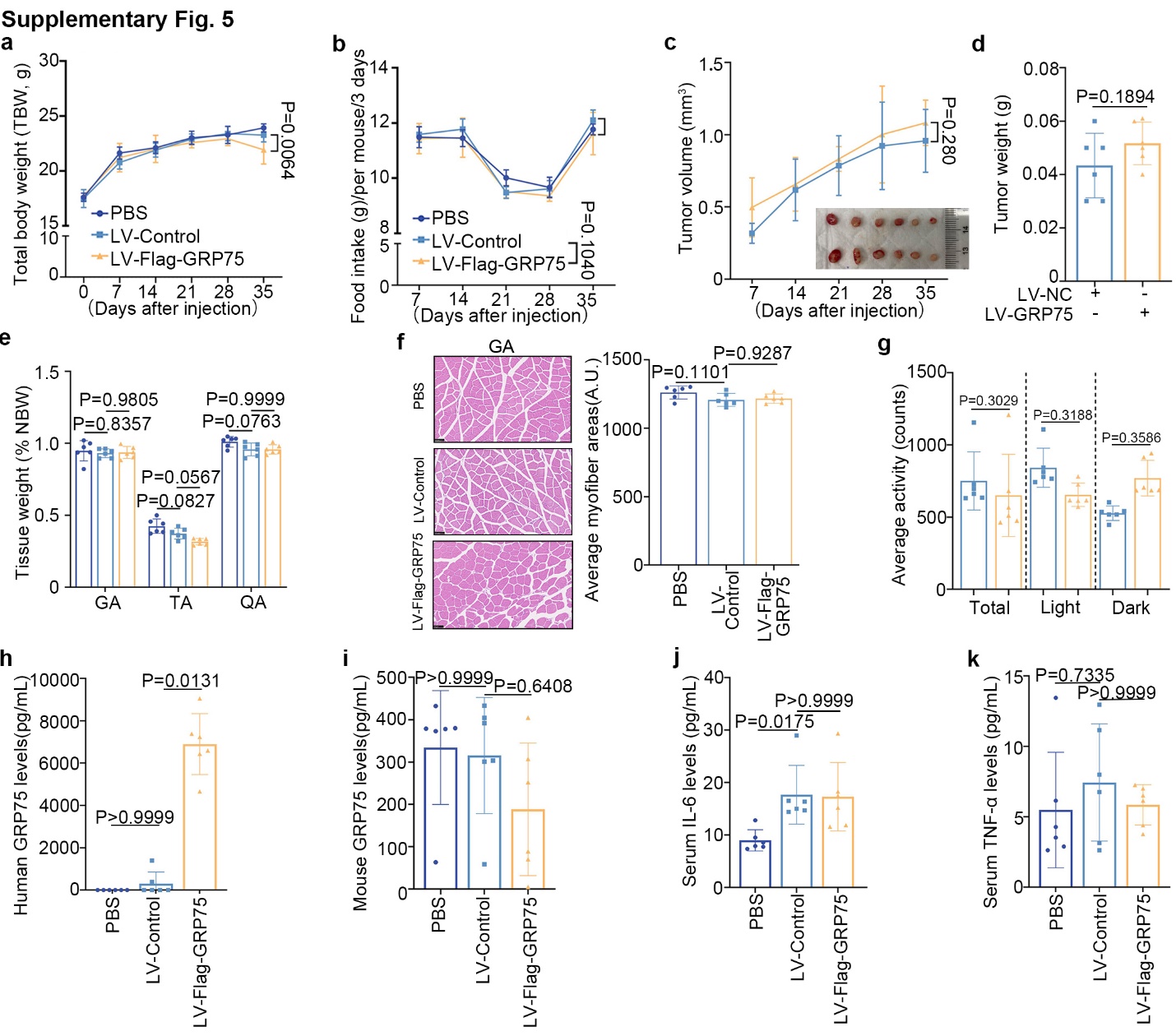


Figure. S5. GRP75 is essential for adipocyte browning

**a**, **b** Total body weight (TBW) and food intake curves of the PBS, LV-Control and LV-Flag-GRP75 groups. **c**, **d** Subcutaneous tumor growth curves (**c**) and tumor weights (**d**) of LV-Control and LV-Flag-GRP75 groups. The image of tumor from two groups was shown in (**c**). **e** Ratios of skeletal muscle (GA, TA, and QA) to NBW in three groups. **f** Representative images of H&E staining in GA muscle from three groups and quantification of myofiber areas is shown in the right side. **g** Average X+Y activity (counts) of LV-Control and LV-Flag-GRP75 groups. **h**–**k** Human GRP75 (**h**), and mouse GRP75 (**i**), IL-6 (**j**) and TNF-α (**k**) levels in serum of three groups were assessed by enzyme linked immunosorbent assay. The data are presented as means ± SEM (n = 6 mice per group). The exact *P* values were tested with two-way ANOVA multiple comparison in (**a**, **b**) and multiple *t*-test in (**c**), unpaired two-tailed Student’s *t*-test in (**d**, **g**) and one-way ANOVA in (**e**, **f**, **h**–**k**).


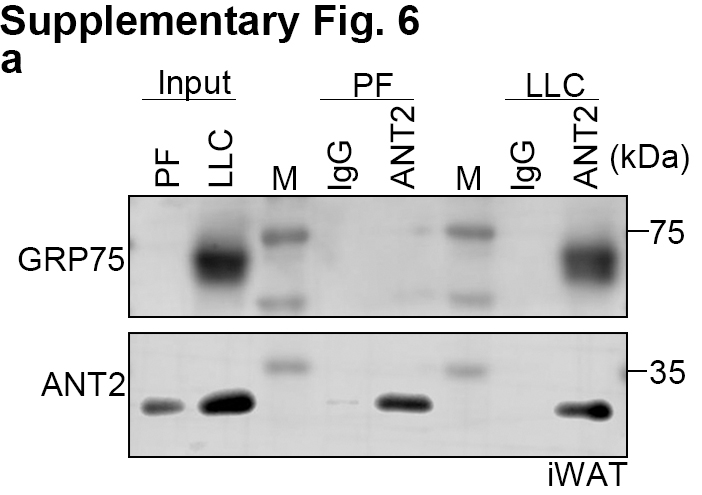


Figure. S6. GRP75 is essential for adipocyte browning

**a** Co-immunoprecipitation of endogenous ANT2 and GRP75 in the iWAT of PF and LLC tumor-bearing groups with anti-ANT2. Anti-IgG was used as a negative control. M, protein marker.


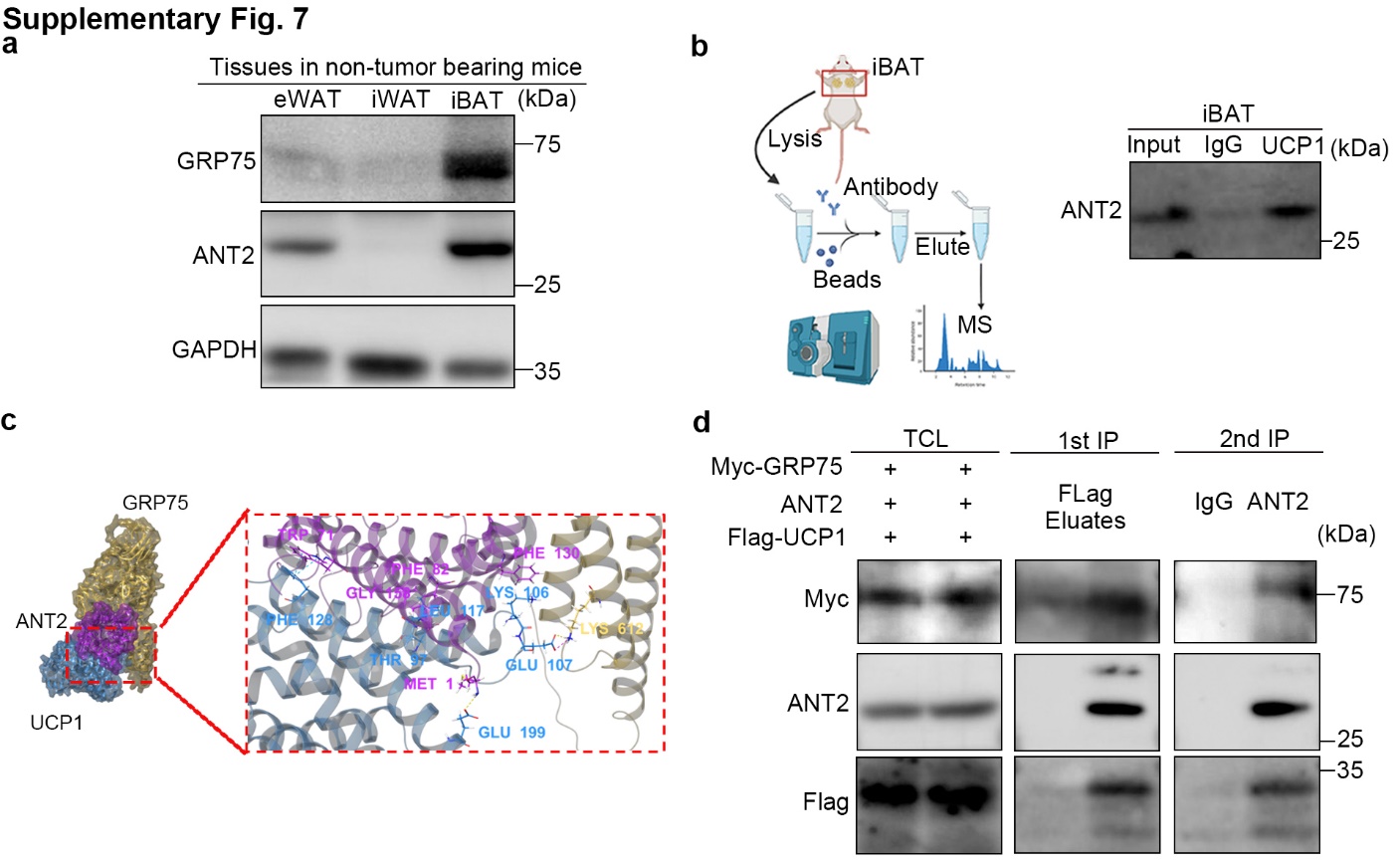


Figure. S7. GRP75 is essential for adipocyte browning

**a** Expression of GRP75 and ANT2 in the BAT of non-tumor bearing mice analyzed by immunoblotting. **b** Schematic diagram for detecting UCP1-binding protein in BAT (left panel) and endogenous co-immunoprecipitation assay of UCP1–ANT2 interactions (right panel). **c** Surface representation of the GRP75–ANT2 complex (yellow and purple) with UCP1 (blue). Stick view showed the most energetically favored pose of GRP75–ANT2–UCP1 in the potential binding sites predicted by Schrodinger Maestro 13.5. The zoomed-in structure displays the interaction details of these proteins. The yellow indicates Hydrogen bonds and the blue indicates π-π bonds. **d** Two-step of co-immunoprecipitation using HEK293T cells transfected with Myc-GRP75, ANT2, and Flag-UCP1. The first round of IP was performed by anti-FLAG antibody followed by elution with the Flag peptide.


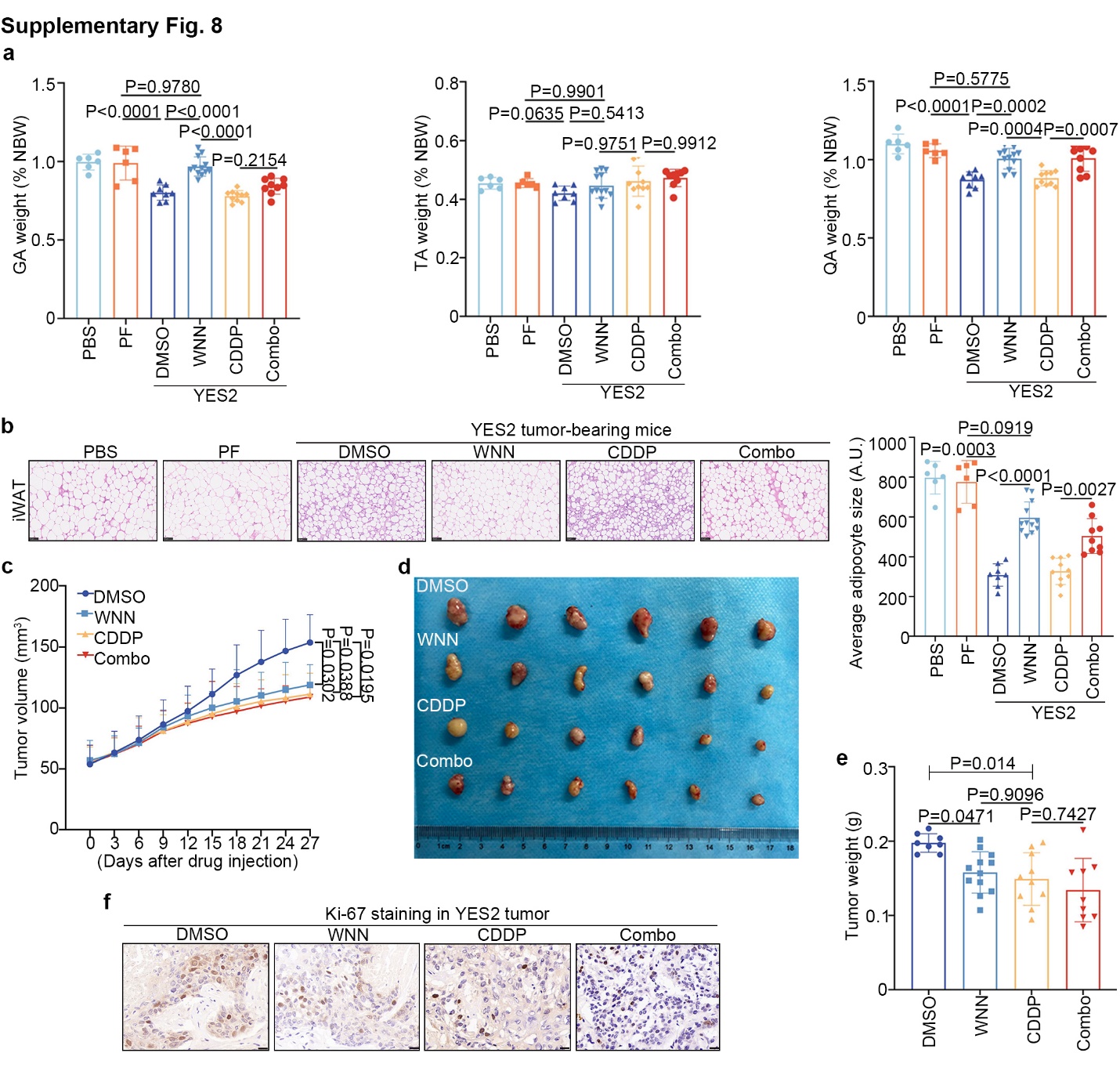


Figure. S8. GRP75 inhibitors alleviate adipocyte browning *in vitro* and *in vivo*

**a** Ratios of skeletal muscles (GA, TA, and QA) to NBW from PBS group, PF group (n = 6 mice/group), YES2 tumor-bearing mice injected with DMSO (DMSO group, n = 8), withanone (WNN group, n = 12), cisplatin (CDDP group, n = 10), or a combination of both (Combo group, n = 9) at day 48. **b** Representative H&E images of iWAT from six groups and quantifications of adipocyte sizes were shown in the right side. Scale bar, 50 μm. **c** Subcutaneous tumor growth curves from DMSO, WNN, CDDP, and Combo groups. **d**, **e** Representative images (**d**) and weights (**e**) of tumors from four groups at day 48 (n = 6; representative of six biological replicates per group). **f** Representative IHC images of Ki-67 expression in tumors shown in (**d**). Scale bar, 50 μm. The data are represented as mean ± SEM. The exact *P* values were tested with one-way ANOVA in (**a**, **b**, and **e**) and two-way ANOVA multiple comparison in (**c**).


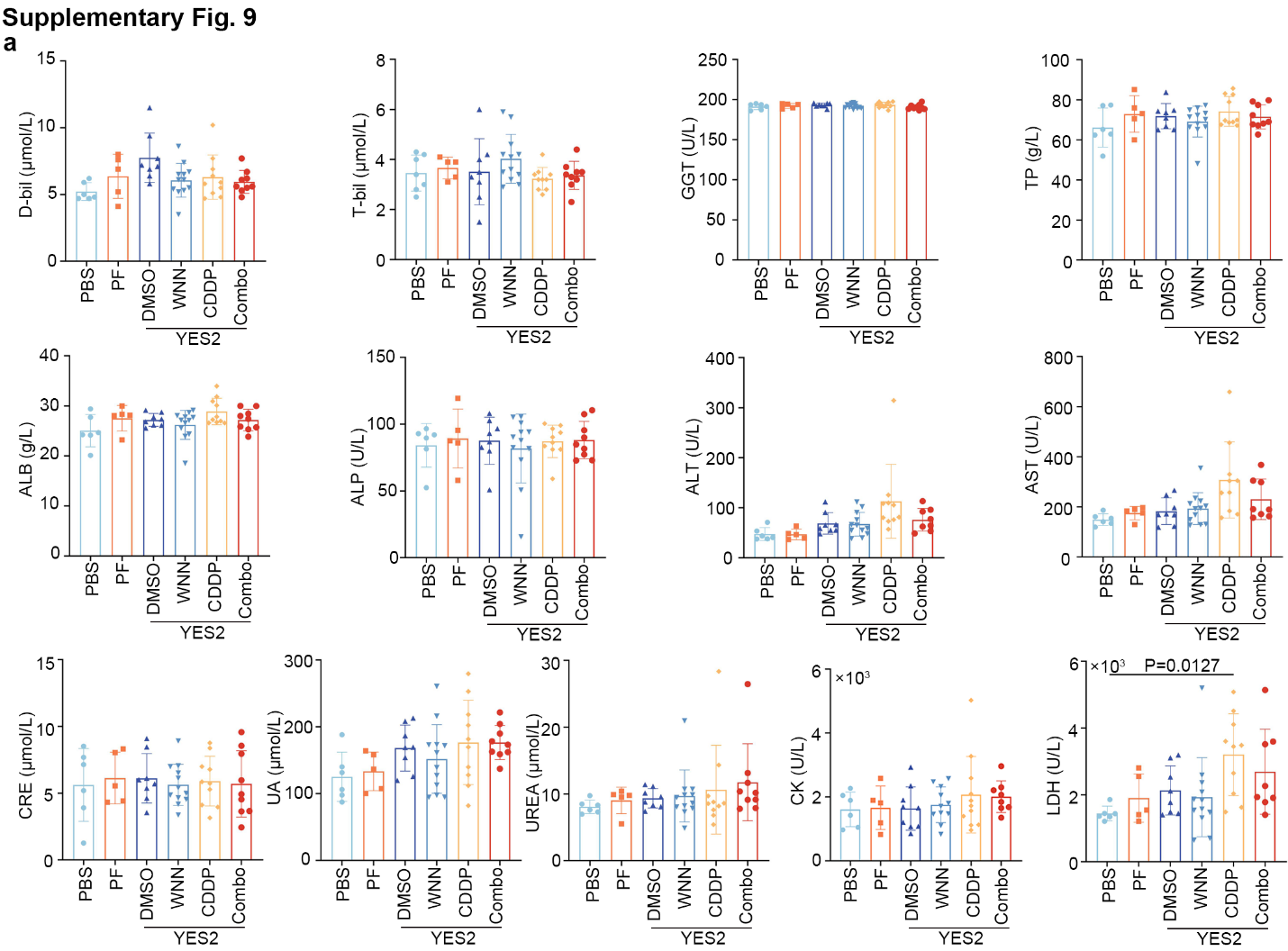


Figure. S9. WNN administration does not cause significant toxic or side effects

**a** Comparative analysis of basic biochemical parameters in sera of six groups. D-bil: direct bilirubin; T-bil: total bilirubin; GGT: γ-glutamyl transpeptidase; TP: total protein; ALB: albumin; ALP: alkaline phosphatase; ALT: alanine aminotransferase; AST: aspartate aminotransferase; CRE: creatinine; UA: uric acid; UREA: creatinine; CK: creatine kinase; LDH: lactate dehydrogenase. The data are represented as mean ± SEM. The exact *P* values were tested with one-way ANOVA in (**a**).

Table S1. Association of GRP75 expression with clinicopathological features of 107 ESCC patients

| **Clinicopathological features** |  | **sum** | **GRP75 Expression** | | **χ2** | **p value** |
| --- | --- | --- | --- | --- | --- | --- |
|  |  |  | **GRP75 low**  **(0**–**8)** | **GRP75 high**  **(9**–**12)** |  |  |
| Gender | male | 81 | 49 | 32 | 2.320 | 0.161 |
|  | female | 26 | 20 | 6 |  |  |
| Age | < 65 | 45 | 30 | 15 | 0.215 | 0.650 |
|  | ≥ 65 | 61 | 38 | 23 |  |  |
| Differentiation degree | I-II | 85 | 51 | 34 | 3.633 | 0.057 |
|  | III | 22 | 18 | 4 |  |  |
| T stage | T1+T2 | 20 | 16 | 4 | 2.365 | 0.124 |
|  | T3 | 81 | 50 | 31 |  |  |
| N stage | N0 | 49 | 38 | 11 | 7.115 | 0.008 |
|  | N+ | 57 | 30 | 27 |  |  |
| TNM stage  (7th AJCC) | I-II | 49 | 41 | 8 | 14.856 | <0.001 |
|  | III | 53 | 25 | 28 |  |  |

**Table S2. The clinicopathological characteristics of 108 ESCC samples.**

| **ID** | **Gender** | **Age** | **T stage** | **N stage** | **TNM stage (7th AJCC)** | **Survival status** | **Time (months)** | **Matched adjacent tissues** |
| --- | --- | --- | --- | --- | --- | --- | --- | --- |
| D08A0181 | Male | 70 | T3 | N0 | 2A | Dead | 58 | YES |
| D08A0184 | Male | 73 | T3 | N1 | 3A | Dead | 26 | YES |
| D08A0185 | Female | 74 | T3 | N+ | 3 | Dead | 13 | YES |
| D08A0187 | Male | 66 | T3 | N0 | 2B | Alive | 107 | YES |
| D08A0188 | Male | 73 | T3 | N0 | 2B | Dead | 15 | NO |
| D08A0194 | Male | 69 | T3 | N1 | 3A | Dead | 6 | NO |
| D08A0196 | Male | 78 | / | N1 | / | Dead | 60 | YES |
| D08A0201 | Male | 67 | T2 | N0 | 2B | Dead | 36 | YES |
| D08A0203 | Male | / | T3 | N1 | 3A | Dead | 18 | YES |
| D08A0205 | Female | 79 | T3 | N2 | 3B | Dead | 8 | YES |
| D08A0209 | Male | 72 | T3 | N1 | 3A | Dead | 5 | NO |
| D08A0211 | Male | 74 | / | N0 | / | Dead | 1 | YES |
| D08A0212 | Male | 78 | T3 | N0 | 2 | Dead | 7 | NO |
| D08A0214 | Male | 29 | T3 | N1 | 3A | Dead | 11 | NO |
| D08A0216 | Male | 84 | T3 | N2 | 3B | Dead | 6 | YES |
| D08A0218 | Male | 74 | / | N2 | 3 | Dead | 6 | YES |
| D08A0219 | Male | 66 | T3 | N1 | 3A | Dead | 2 | NO |
| D08A0221 | Male | 50 | / | N0 | / | Dead | 2 | NO |
| D08A0256 | Male | 83 | T4 | N0 | 3 | Dead | 18 | YES |
| D08A0257 | Male | 65 | T3 | N0 | 2 | Dead | 4 | NO |
| D08A0260 | Male | 75 | T3 | N0 | 2 | Dead | 31 | YES |
| D08A0284 | Male | 51 | T3 | N0 | 2B | Dead | 51 | YES |
| D08A0285 | Male | 51 | T3 | N2 | 3B | Dead | 47 | NO |
| D08A0286 | Male | 73 | / | N0 | / | Dead | 29 | YES |
| D08A0290 | Male | 63 | T3 | N1 | 3A | Dead | 22 | NO |
| D08A0293 | Male | 51 | T3 | N2 | 3B | Dead | 35 | YES |
| D08A0294 | Male | 60 | T3 | N2 | 3B | Dead | 10 | NO |
| D08A0334 | Male | 72 | T3 | N2 | 3B | Dead | 2 | YES |
| D08A0336 | Female | 62 | T2 | N0 | 2B | Alive | 97 | NO |
| D08A0337 | Male | 71 | T3 | N2 | 3B | Dead | 23 | YES |
| D08A0340 | Male | 51 | T2 | N0 | 2B | Dead | 9 | YES |
| D08A0373 | Male | 64 | T3 | N3 | 3C | Dead | 6 | NO |
| D08A0376 | Male | 64 | T3 | N0 | 2B | Dead | 43 | YES |
| D08A0380 | Male | 52 | T3 | N1 | 3A | Dead | 8 | YES |
| D08A0381 | Male | 59 | T3 | N3 | 3C | Dead | 9 | YES |
| D08A0383 | Male | 70 | T3 | N1 | 3A | Dead | 16 | YES |
| D08A0384 | Male | 65 | T3 | N0 | 2A | Alive | 97 | YES |
| D08A0385 | Male | 63 | T2 | N0 | 2B | Dead | 26 | YES |
| D08A0388 | Female | 56 | T3 | N0 | 2B | Alive | 97 | YES |
| D08A0391 | Male | 73 | T3 | N0 | 2B | Dead | 33 | NO |
| D08A0394 | Male | 51 | T3 | N0 | 2B | Dead | 6 | NO |
| D08A0395 | Male | 49 | T3 | N2 | 3B | Dead | 8 | NO |
| D08A0400 | Male | 48 | T3 | N1 | 3A | Dead | 43 | NO |
| D08A0401 | Male | 53 | T3 | N1 | 3A | Dead | 44 | YES |
| D08A0402 | Female | 71 | T3 | N0 | 2A | Alive | 95 | YES |
| D08A0403 | Male | 69 | T3 | N1 | 3A | Dead | 23 | YES |
| D08A0404 | Male | 61 | T3 | N1 | 3A | Alive | 72 | YES |
| D08A0405 | Male | 77 | T3 | N0 | 2 | Dead | 4 | NO |
| D08A0477 | Male | 61 | T3 | N1 | 3A | Dead | 9 | NO |
| D08A0510 | Male | 65 | T3 | N0 | 2 | Dead | 12 | NO |
| D08A0514 | Male | 53 | T3 | N1 | 3A | Dead | 13 | YES |
| D08A0515 | Male | 66 | T2 | N0 | 2B | Dead | 50 | YES |
| D08A0548 | Female | 63 | T1 | N0 | 1 | Dead | 41 | NO |
| D08A0569 | Male | 59 | T3 | N0 | 2 | Dead | 13 | NO |
| D08A0571 | Female | 56 | T3 | N0 | 2A | Dead | 15 | YES |
| D08A0572 | Female | 68 | T3 | N0 | 2 | Dead | 13 | YES |
| D08A0577 | Male | 57 | T2 | N1 | 2B | Alive | 88 | YES |
| D08A0579 | Male | 61 | T3 | N0 | 2 | Dead | 33 | YES |
| D08A0580 | Male | 68 | T3 | N3 | 3C | Dead | 12 | NO |
| D08A0581 | Female | 72 | T3 | N0 | 2A | Dead | 12 | YES |
| D08A0582 | Male | 57 | T3 | N0 | 2A | Alive | 88 | NO |
| D08A0583 | Male | 74 | T3 | N0 | 2B | Dead | 4 | YES |
| D08A0584 | Male | 72 | T2 | N2 | 3A | Dead | 5 | YES |
| D08A0588 | Female | 65 | T3 | N0 | 2B | Dead | 30 | YES |
| D08A0589 | Male | 65 | T3 | N1 | 3A | Dead | 15 | NO |
| D08A0593 | Male | 75 | T3 | N0 | 2B | Alive | 87 | YES |
| D08A0594 | Female | 58 | T3 | N0 | 2 | Alive | 87 | NO |
| D08A0595 | Male | 80 | T3 | N1 | 3A | Dead | 5 | NO |
| D08A0596 | Female | 63 | T3 | N0 | 2B | Dead | 22 | NO |
| D08A0648 | Female | 73 | T3 | N2 | 3B | Dead | 5 | YES |
| D08A0649 | Male | 76 | T3 | N0 | 2 | Dead | 0 | YES |
| D08A0650 | Male | 61 | T3 | N1 | 3A | Dead | 5 | NO |
| D08A0651 | Female | 57 | T3 | N1 | 3A | Alive | 87 | NO |
| D08A0652 | Female | 79 | T3 | N0 | 2B | Alive | 86 | YES |
| D08A0655 | Female | 71 | T3 | N0 | 1B-2 | Dead | 6 | NO |
| D08A0659 | Male | 50 | T3 | N2 | 3B | Dead | 7 | NO |
| D08A0660 | Male | 54 | T2 | N2 | 3A | Dead | 14 | NO |
| D08A0692 | Male | 79 | T2 | N3 | 3C | Dead | 15 | YES |
| D08A0694 | Male | 72 | T3 | N1 | 3A | Dead | 5 | YES |
| D08A0696 | Male | 54 | T3 | N1 | 3A | Dead | 16 | YES |
| D08A0697 | Male | 76 | T2 | N1 | 2B | Alive | 84 | YES |
| D08A0699 | Male | 65 | T3 | N3 | 3C | Dead | 26 | NO |
| D08A0700 | Female | 81 | T2 | N0 | 2B | Alive | 84 | NO |
| D08A0701 | Female | 68 | T3 | N1 | 3A | Alive | 84 | YES |
| D08A0702 | Male | 81 | T3 | N1 | 3A | Dead | 1 | YES |
| D08A0705 | Female | 74 | T3 | N0 | 2A | Alive | 83 | YES |
| D08A0718 | Male | 64 | T3 | N1 | 3A | Dead | 5 | YES |
| D08A0719 | Female | 71 | T1 | N0 | 1 | Alive | 83 | YES |
| D08A0720 | Male | 62 | T3 | N2 | 3B | Dead | 4 | NO |
| D08A0723 | Female | 68 | T3 | N1 | 3A | Dead | 25 | YES |
| D08A0724 | Male | 74 | T3 | N2 | 3B | Dead | 10 | NO |
| D08A0745 | Male | 79 | T3 | N1 | 3A | Dead | 27 | NO |
| D08A0746 | Female | 74 | T1 | N0 | 1 | Alive | 82 | YES |
| D08A0748 | Male | 63 | T1 | N0 | 1 | Alive | 81 | YES |
| D08A0752 | Male | 65 | T3 | N1 | 3A | Dead | 28 | NO |
| D08A0753 | Male | 52 | T3 | N1 | 3A | Dead | 10 | YES |
| D08A0803 | Male | 52 | T2 | N1 | 2B | Alive | 83 | NO |
| D08A0804 | Female | 74 | T3 | N0 | 2 | Dead | 10 | NO |
| D08A0807 | Female | 69 | T2 | N0 | 2B | Dead | 70 | NO |
| D08A0834 | Male | 77 | T2 | N0 | 2B | Dead | 58 | NO |
| D08A0835 | Male | 69 | T2 | N1 | 2B | Alive | 79 | YES |
| D08A1772 | Male | 64 | T3 | N1 | 3A | Dead | 31 | YES |
| D08A1794 | Male | 56 | / | / | / | Dead | 30 | YES |
| D08A1820 | Male | 62 | T3 | N1 | 3A | Dead | 11 | YES |
| D08A1822 | Male | 59 | T3 | N2 | 3B | Dead | 12 | NO |
| D08A1837 | Male | 73 | T2 | N0 | 2B | Dead | 88 | YES |
| D08A1843 | Female | 56 | T3 | N0 | 2 | Alive | 79 | YES |
| D08A1844 | Female | 51 | T3 | N1 | 3A | Dead | 41 | NO |

**Table S3. Identification of protein binding to GRP75 by mass spectrometry**

| Number | Protein | | Unique peptides |
| --- | --- | --- | --- |
| 1 | | Myosin-9 | 23 |
| 2 | | Vimentin | 15 |
| 3 | | Junction plakoglobin | 13 |
| 4 | | 78 kDa glucose-regulated protein | 22 |
| 5 | | Poly [ADP-ribose] ploymerase 1 | 10 |
| 6 | | Heat shock cognate 71 kDa protein | 8 |
| 7 | | Desmoplakin | 7 |
| 8 | | Plasminogen activator inhibitor 1 RNA-binding protein | 7 |
| 9 | | ADP/ATP translocase 2 | 7 |

Data S1. Western blot bands


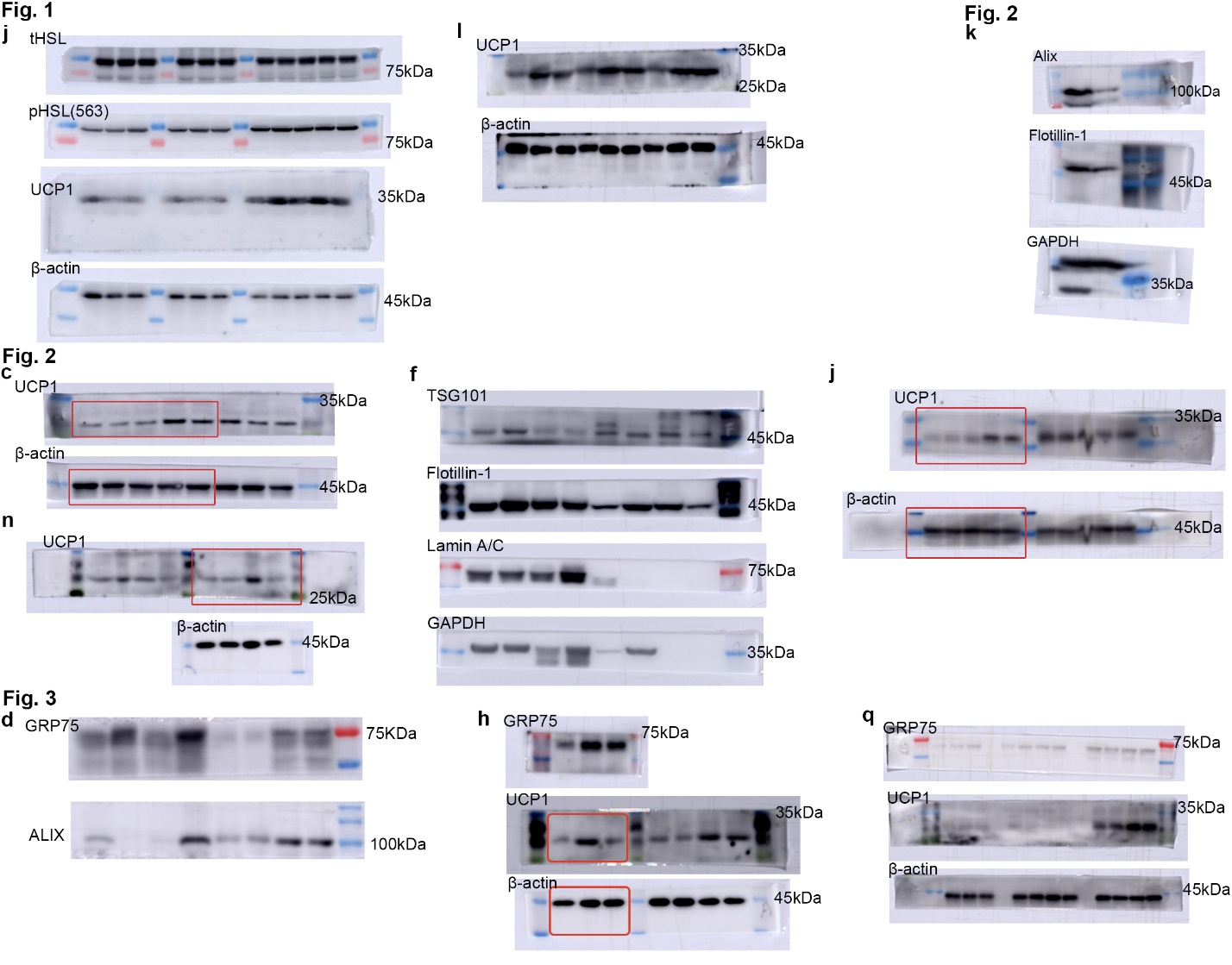


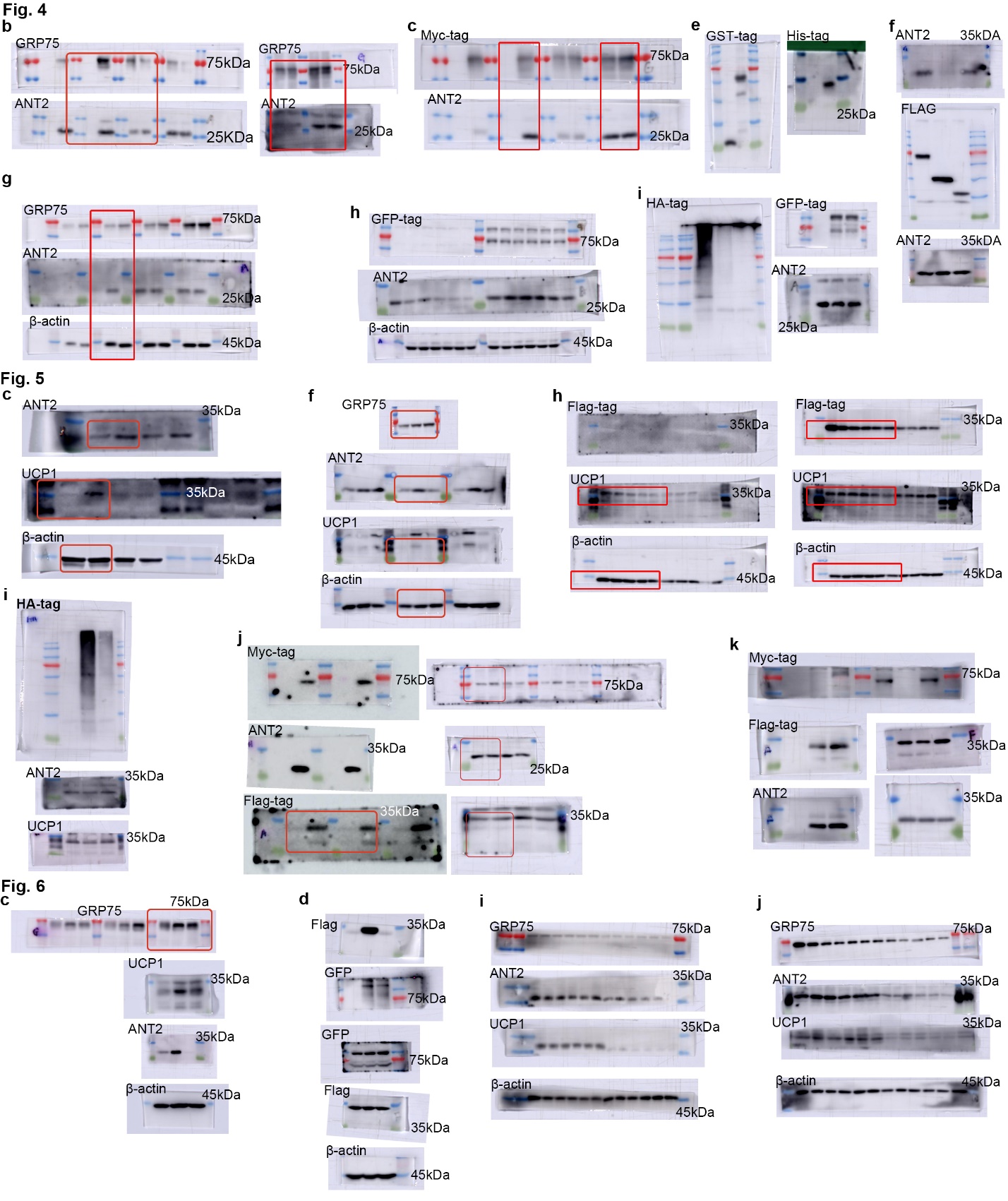


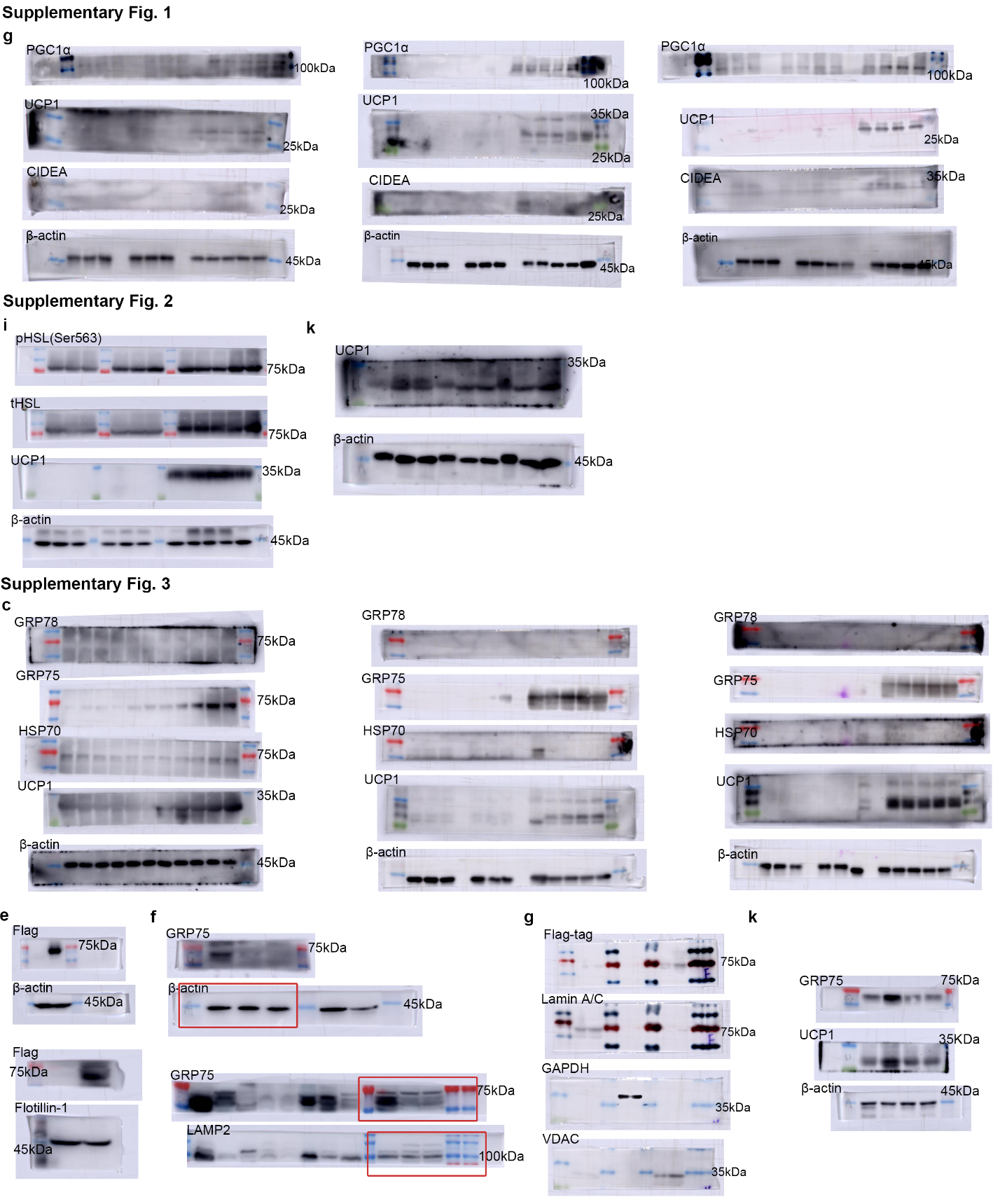


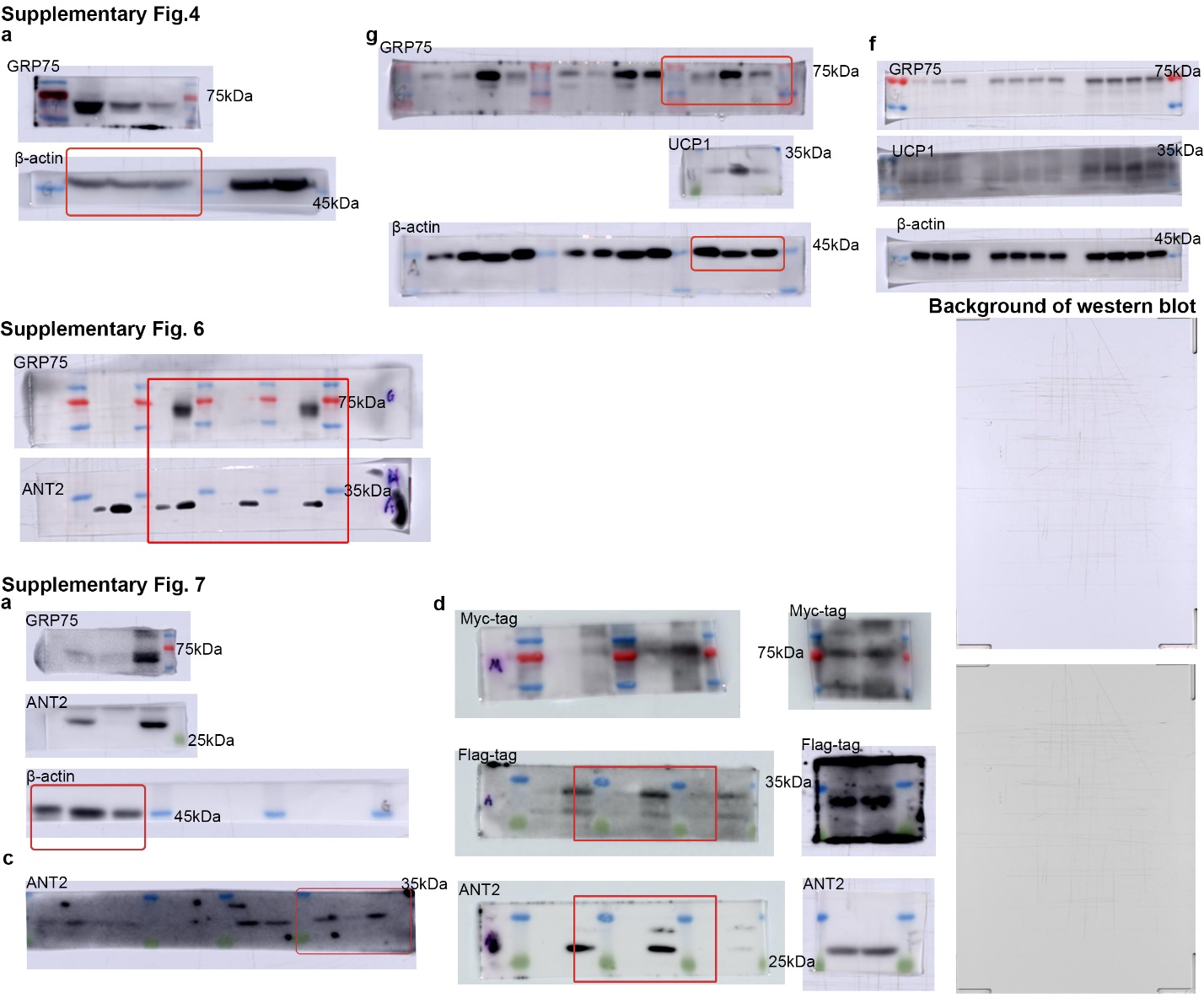


Data S2. Ethical Approval Form for Immunohistochemistry Chips in Human Esophageal Squamous Cell Carcinoma


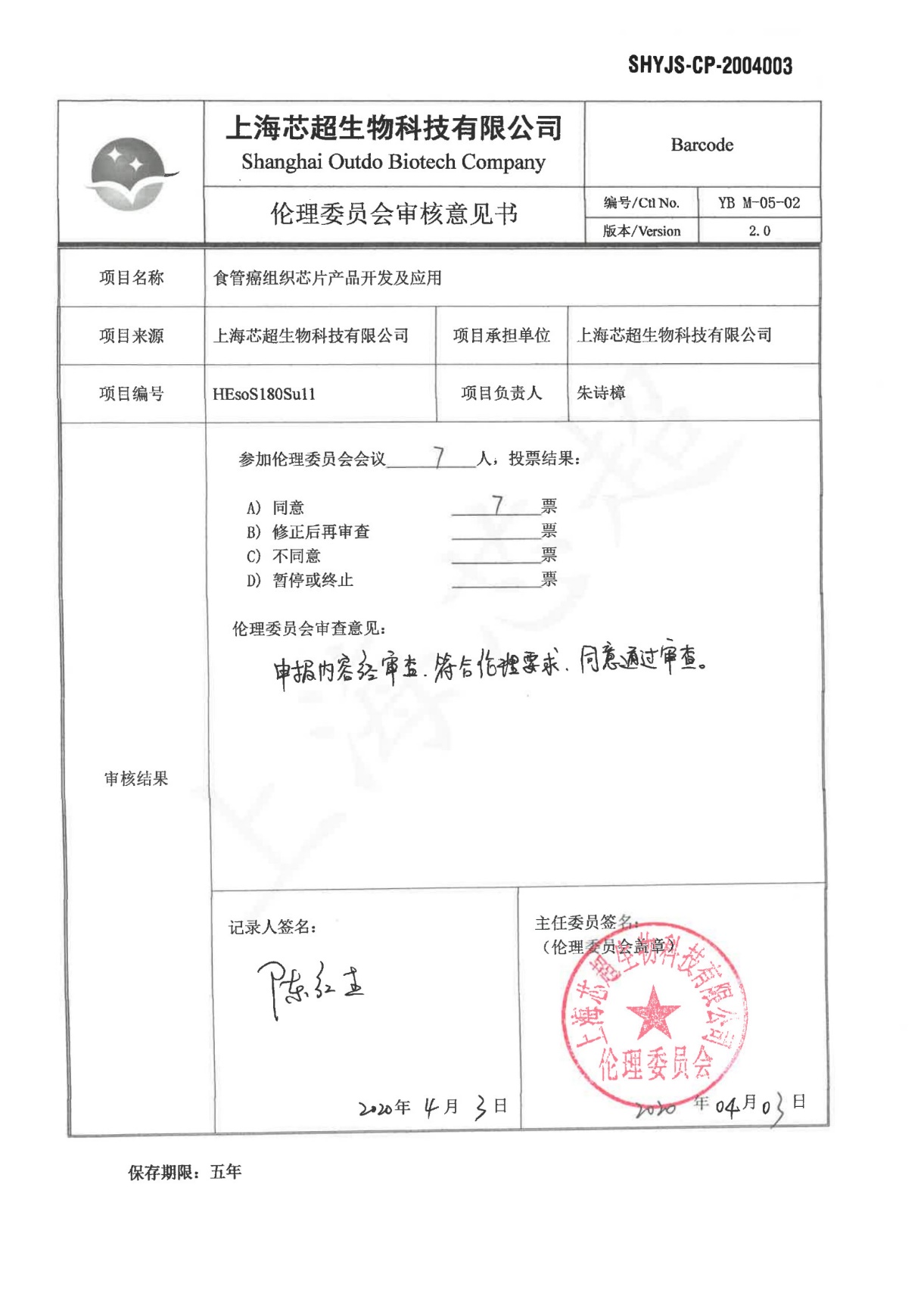

Supplement: Supplementary file 1 — Supplementary_Materials [file 41392_2024_1950_MOESM1_ESM.docx]
